# Engineering metal‐organic framework‐based smart porous nanoplatforms for targeted therapy and theranostic applications

**DOI:** 10.1002/smo2.70113

**Published:** 2026-10-08

**Authors:** Jagabandhu Bag, Subhasis Banerjee, Sabyasachi Banerjee, Sourav De, S. K. Ashok Kumar, Yan‐Jye Shyong, Subhankar Mukhopadhyay

**Affiliations:** ^1^ Institute of Clinical Pharmacy and Pharmaceutical Sciences College of Medicine National Cheng Kung University Tainan Taiwan; ^2^ Department of Pharmaceutical Technology Eminent College of Pharmaceutical Technology Kolkata West Bengal India; ^3^ Department of Pharmaceutical Chemistry Gupta College of Technological Sciences Asansol West Bengal India; ^4^ Department of Chemistry School of Advanced Sciences Vellore Institute of Technology Vellore India; ^5^ The Jockey Club School of Public Health and Primary Care Faculty of Medicine The Chinese University of Hong Kong Hong Kong China; ^6^ System Microbiology and Antimicrobial Resistance (SMART) Lab Li Ka Shing Institute of Health Sciences Faculty of Medicine The Chinese University of Hong Kong Hong Kong China

**Keywords:** absorption, distribution, metabolism, and excretion, diagnostic, metal–organic frameworks, porous nanoplatforms, theranostic, therapeutic

## Abstract

Metal‐organic frameworks (MOFs) are materials that have recently emerged with the potential for various biomedical applications. Through engineering MOFs to contain various components and functionalities, they have progressed from porous materials to “smart” MOFs that can deliver payloads to cells, release the payloads in specific situations, perform certain therapies on the cells, and even perform diagnosis and therapy procedures simultaneously. This review aims to highlight the synergistic induction of ferroptosis through nanomedicine, immunotherapy, and targeted therapeutic agents for the treatment of breast and gastrointestinal (GI) malignancies. Nanotechnology‐based targeted delivery systems enhance the delivery of ferroptosis‐inducing agents to cancer cells while minimizing the toxicity of these agents to healthy cells. Additionally, immunotherapeutic agents enhance the immune response to cancer cells and sensitize them to ferroptotic cell death. Targeted therapeutic agents that act on the molecular regulators of ferroptosis, such as GPX4, System Xc^−^, NRF2, and iron metabolism, potentiate ferroptosis. The combination of these therapeutic strategies has the potential to reverse multidrug resistance, limit metastasis, and increase the efficacy of cancer therapy. Furthermore, this review discusses the current advances in and future perspectives on the development of combination therapies targeting ferroptotic cell death in cancer cells. The integration of nanomedicine, immunotherapy, and targeted therapeutic agents presents a highly promising new avenue for treating breast and gastrointestinal cancers.

## Graphical abstract

MOFs offer high porosity and tunability for biomedical applications, especially in cancer therapy via ROS generation and targeted delivery. Though toxicity, stability, and scalability remain key clinical challenges. Drug loading in MOFs exceeds 60%–70% with controlled release behavior.

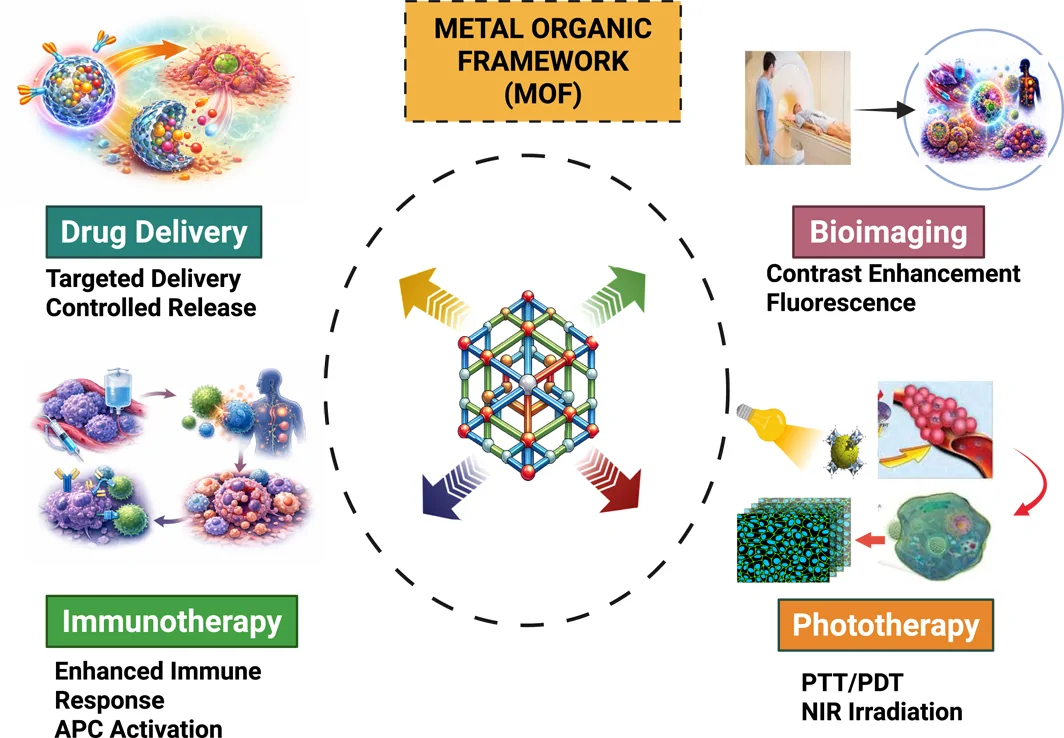

## 1 INTRODUCTION

Generally, therapeutic biomaterials are associated with several medicinal applications with many typical limitations, such as side effects, low bioavailability, and rapid systemic clearance. To mitigate these issues and maintain the consistency of the drug delivery system, several nanoparticles (NPs) are used for potential application with better bioavailability, high entrapment efficiency, improved pharmacokinetics mechanism, targeted delivery, and many advantages due to their smaller size and high surface area. Furthermore, NPs offer a non‐invasive platform with long‐term monitoring for diagnostic and bioimaging purposes. As a result, several other forms of NPs have been developed, including polymeric NPs, liposomes, polymeric micelles, magnetic NPs, and dendrimers. Each nanomaterial has advantages and disadvantages in terms of stability, toxicity, drug loading capability, and other features.[^1^, ^2^, ^3^] In this context, metal‐organic frameworks (MOFs) are an intriguing type of crystalline porous materials made up of different metal ions and organic linkers.

MOFs are promising alternatives in many applications due to their exceptionally high porosity, surface area, large pore size, intrinsic biodegradability, biocompatibility, and versatile potential functionality for post‐synthetic modifications.^[4]^ Metal‐organic frameworks (MOFs) are highly porous and stable crystalline materials that can have one‐, two‐ or three‐dimensional structures and contain metal ions or clusters that are coordinated with organic ligands. Since the introduction of MOFs in the 1990s, there have been significant developments in the field, allowing for the creation of MOFs of various structures.^[5]^ The ability of MOFs to utilize various metal nodes and organic linkers allows the creation of a variety of MOF structures with the potential for functionalization. Furthermore, MOFs can be categorized into various types of MOFs, each with different structures and properties, such as zeolitic imidazolate frameworks (ZIFs), isoreticular MOFs (IRMOFs), covalent organic frameworks (COFs), and porous coordination polymers (PCPs).^[6]^ The modular nature of MOFs provides control over the size of the pores within the MOF, the chemistry of the pores, the stability of the MOFs, the degradability of the MOFs, and how the MOFs interact with the molecules to be delivered. These factors are crucial to be able to control for the various objectives of MOFs in biomedicine. The synthesis process of MOF can be accomplished through a variety of techniques, such as solvothermal, hydrothermal, microwave‐assisted, electrochemical, and sonochemical methods.[^7^, ^8^] The structural properties of MOFs and their derivatives can be modified using four major strategies: (1) customizing their metal centres and ligands, (2) reconfiguring their morphology, (3) engineering MOF‐based hybrid materials, and (4) synthesising MOF‐derived functional molecules.

To determine their performance in effective application in various fields, characteristics, such as composition and structural configuration, are important.[^9^, ^10^] An extensive study has been conducted on the essential structures of MOFs due to their outstanding properties, such as high specific surface area (SSA) and porosity. These structures with their changeable porosity offer an adaptable platform for research in various fields. MOFs' unique nano‐ and micro‐scale properties, including higher porosity, larger surface area, changeable pore size, biodegradability, bio‐compatibility, as well as responsiveness to post‐synthesis changes, make them interesting candidates for a wide range of applications. In recent years, significant progress has been achieved in the structural design of MOFs to enhance their functional diversity.^[11]^ Different in‐process strategies are followed to maintain the structure of this composite, such as multivariate synthesis (the incorporation of numerous linkers or metals into a single framework), post‐synthetic modification, and topological control. The development of hierarchical porous MOFs, flexible and dynamic frameworks, and stimuli‐responsive materials has expanded the spectrum of their applications.^[12]^ The most intriguing applications of MOFs in the medical field include their potential use in bone regeneration, drug delivery, and theranostic systems. Due to the dual effect that MOFs have on the inside and outside of biological systems, MOFs have recently played a crucial role in the creation of theranostic systems for a variety of diseases and medical conditions.[^12^, ^13^] Furthermore, researchers are interested in the biological potential of MOFs, especially in drug delivery and bioimaging. Their unique structure can encapsulate various biomaterials to target a specific cell or tissue.^[4]^ In addition, the emergence of nanostructured and nanoscale MOFs (NMOFs) has introduced high porosity, large surface area, and versatile characteristics, making them excellent for targeted drug administration with controlled‐release mechanisms. However, despite these exceptional capabilities, there are various hurdles that impede their clinical application.^[14]^

One of the major challenges associated with their accumulation in the body, which is caused by the presence of heavy metal nodes, is another issue regarding their drug loading and release profile. However, in recent years, substantial research has been conducted to address these concerns. Another important issue to consider is the particle size of MOFs, which influences bio‐distribution, bio‐compatibility, circulating time, and in vivo elimination. As a result, particle size regulation and maintaining size have emerged to improve applications in biomedicine and many medical fields.^[15]^ In recent years, micro‐ and nano‐scale methods have gained importance and popularity in regenerative medicine. In addition to the cancers that have been discussed in relation to the relevant engineering capabilities of MOFs, the study of breast cancer and gastrointestinal (GI) malignancies also provides settings in which those capabilities can be investigated. Each of these tumor types is composed of biologically heterogeneous tumors that may exhibit limitations to the efficacy of conventional cancer treatments. For these reasons, conventional drugs may not exhibit satisfactory therapeutic windows for the treatment of cancer patients with these types of tumors.^[16]^ Thus, an engineered drug delivery system that can incorporate the various functionalities of MOFs may offer advantages over conventional drug formulations. Thus, each of these tumor types was selected for discussion in this review in relation to the capabilities of MOFs, and not based upon the assumption that the tumors of these particular cancer types will exhibit an intrinsic and exclusive response to MOFs.^[17]^

The advantages of MOFs in these tumor settings arise from their engineering potential. MOFs can be engineered to incorporate various types of therapeutics, such as chemotherapeutic agents, nucleic acids, and photosensitizers. The metal nodes that form the MOFs can be engineered to be stable or to degrade under certain conditions, as can the organic linkers that form the MOFs. Additionally, the MOFs can be functionalized with targeting ligands, such as folic acid, transferrin, antibodies, aptamers, hyaluronic acid, or other biomimetic components that will enhance the targeting of the MOFs to the tumor cells. Moreover, MOFs can be engineered to respond to certain tumor or intracellular components, such as acidic pH, redox components, reactive oxygen species (ROS), glutathione, hypoxia, ATP, or enzymes. Such engineering allows for the shift in drug release from predominantly passive delivery to targeted and responsive drug delivery. Each of these features is particularly relevant to tumors wherein the delivery of the agents into the tumor cells is important and wherein the reduction of the toxicity of those agents to healthy cells is desirable.^[18]^ The breast cancer literature includes several examples of MOFs that take advantage of these engineering features. For instance, folic acid‐functionalized MOFs have been investigated that can deliver the chemotherapeutic agent bufalin in response to acidic and redox components of the tumor cells, and have experienced enhanced antitumor activity in comparison to the free chemotherapeutic agent.^[19]^ Furthermore, folic acid‐conjugated UiO‐66 MOFs have been investigated for the co‐delivery of the chemotherapeutic agents cisplatin and cyclophosphamide to breast cancer cells, during which time the agents were encapsulated into the MOFs and delivered to the breast cancer cells, resulting in enhanced apoptotic responses in the breast cancer cells and a reduction in their cancer cell invasion 90. Additionally, transferrin‐functionalized PEGylated MOF‐74 has been investigated for the targeted delivery of doxorubicin to triple‐negative breast cancer cells, targeting the transferrin receptor that is overexpressed on those cancer cells.^[20]^ These are but a few of the many examples of the advantages of utilizing MOFs for targeted delivery of therapeutics to breast cancer cells.

Similar principles are relevant to GI malignancies. Furthermore, there are other examples of MOF‐based systems that have employed the acidic nature of the cellular environment or the characteristics of the tumor microenvironment to regulate the release of their cargo or to lead to its degradation. For instance, MOF‐303 has been investigated as a system that can target liver cancer cells, utilizing the ability of MOF‐303 to localize to the endosomal and lysosomal compartments within the cancer cells, and the subsequent release of its payload within the acidic compartments of those cells 89. Additionally, a biomimetic system composed of Zr‐MOF that contained a photosensitizer and siHIF‐1α has been investigated as a system that can target esophageal cancer cells, with studies revealing that cancer cells took up the MOF particles, were exposed to light to release ROS to induce apoptosis in the cancer cells, and suppressed HIF‐1α to inhibit tumor growth.^[21]^ Thus, these strategies indicate that MOFs can be utilized in methods beyond chemotherapy. Importantly, the evidence indicates that there is no biological response to MOFs that is limited to breast or GI tumors. The advantage of MOFs is their adaptability to the characteristics of the tumor that is to be targeted. For example, breast tumors may contain receptors for folate and transferrin, and GI tumors may contain acidity within the cells, redox imbalance, hypoxia within the tumors, biomarkers, and various other characteristics that can be exploited by MOFs that are engineered according to the characteristics of that specific tumor.^[22]^ Thus, the focus of this review is on engineering MOFs to take advantage of these tumor characteristics, rather than attributing tumor‐specific properties to the MOF material itself.

Several micro/nanomaterials, including particles, composites, and tailored surfaces, have offered novel approaches for bone tissue regeneration that incorporate a variety of advanced methodologies.^[23]^ MOFs have become one of the fastest‐growing classes of biomaterials for biomedical applications. Numerous review articles have explored the synthesis, physicochemical characteristics, drug‐delivery properties, and biomedical applications of MOFs.[^24^, ^25^, ^26^, ^27^] While there is a wealth of knowledge published on MOFs to date, the field is rapidly moving beyond the synthesis of these materials to engineering multifunctional MOFs for biomedical applications. More recently, MOFs have been engineered to function as smart porous nanoplatforms with a variety of incorporated functions. These intelligent platforms incorporate various elements such as programmable chemistry within the pores of the MOFs, stimuli‐responsive degradation, biomimetic materials, targeting ligands, immune system modulation, and theranostic functions. The engineering of these MOFs is therefore also becoming as important as their use for specific biomedical applications. While there are a variety of reviews on various aspects of MOF technology, the majority of these reviews focus on synthetic methodologies, structural aspects of MOFs, therapeutic applications of MOFs, or theranostics specifically. Fewer reviews exist that focus on the engineering aspects of these MOFs and how they impact the clinical aspects of the MOFs. Furthermore, there have been a growing number of studies regarding MOFs that incorporate various multifunctional aspects (such as stimuli‐responsive aspects, biomimetics, MOF structures, and safety engineering), but there are few reviews that discuss these various aspects as they relate to each other.

Accordingly, this review article has an engineering‐centred perspective that goes beyond the conventional discussion of the applications of MOFs. The applications of MOFs are not discussed individually in this article, but rather, the structural engineering of MOFs is discussed as it relates to the development of intelligent MOFs that can be used in biomedical applications. Furthermore, knowledge regarding the ADME properties of MOFs, their interactions with the immune system, safety, regulatory, and clinical considerations is also integrated into this review to provide a more comprehensive discussion of the topic from engineering to future implementation of these smart MOFs in biomedical systems. Thus, this review aims to highlight the engineering principles that will shape the next generation of smart porous MOFs for biological and medical applications.

Within the framework of these topics, this review aims to specifically examine engineered MOFs for targeted therapy and theranostic applications in the context of breast cancer and GI malignancies. Rather than provide a general review of the ways in which MOFs can be utilized for cancer therapy and theranostics, this review will discuss the impact of engineering these MOF structures in different ways to provide the desired properties for cancer therapy and theranostics. Such discussions will focus on the ability of these engineered structures to target tumors, provide targeted delivery of therapy to those tumors, release the therapy at the tumors, and provide combined or simultaneous therapeutic and diagnostic functions. Additionally, discussions will include the impact of these properties on the ADME behaviour, interaction with the immune system, biosafety, biodegradability, and manufacturing requirements of these MOF nanoparticles. The focus on breast and GI malignancies therefore allows for the review of MOF engineering in a disease‐centered manner. The cancers discussed in this paper are not described as the sole targets for MOF‐based therapies, but are discussed as only a few of the cancers that can be evaluated for the role of MOFs in treating those cancers. Overall, then, the review aims to discuss each of the cancers in relation to the engineering of the MOFs to provide an understanding of which strategies are best for MOF engineering for cancer treatment, which strategies are still lacking in the preclinical literature, and which challenges must be overcome before such smart MOF nanoparticles can be introduced as clinical treatments for cancer patients.

## 2 EVOLUTION OF MOFs: FROM POROUS FRAMEWORKS TO SMART BIOMEDICAL NANOPLATFORMS

Prussian blue, a well‐known metal ligand coordination complex, was first introduced in the 18th‐century. Tomic was the first to explore such materials with metal‐organic architectures, and the first to study the thermal stability of coordinated polymers from selected ligands and metal ions.^[28]^ These early‐aged materials furnished the pathway for the creation of modern MOFs. Furthermore, Hoskins et al. studied more, and the research significantly advanced the understanding of MOF.^[29]^ In the late 20th century, activated carbon and zeolites were the most commonly used porous materials.^[30]^ However, their intrinsic structural limitations prevented them from being fully utilized in biological applications and completely removing pollutants from the aquatic environment. As a result, Yaghi and colleagues developed a class of novel composite materials with enhanced regenerative potential and structural integrity.^[31]^ During synthesis, these materials were identified by various forms, including isoreticular MOFs,^[32]^ microporous coordinated polymeric materials,^[33]^ porous coordinated networks (PCN),^[34]^ and zeolite‐like MOFs,^[35]^ as a universally agreed nomenclature was not yet established, since there was no single naming method in use at the time.

Intense research has introduced about 70 newly synthesized MOFs for specialized applications.^[36]^ Research has focused on creating robust and porous MOFs, such as MOF‐74 and MOF‐5, etc.[^37^, ^38^] Additionally, Materials Institute Lavoisier (MIL) MOFs such as MIL‐100, MIL‐96, MIL‐53, etc., PCN MOFs such as PCN‐515,[^39^, ^40^] PCN‐222, and PCN‐12,^[41]^ and ZIFs (ZIF‐67, ZIF‐8, and ZIF‐7)[^42^, ^43^]; and UiO MOFs, such as UiO‐66 and UiO‐67.^[44]^ The remarkable qualities of the aforementioned MOF types are ascribed to their inventive metal and organic precursors, which make them ideal for a variety of industrial uses, such as environmental and biomedical.^[45]^ There has been a significant revolution in current research over the past 20 years, focusses on developing heterogeneous MOFs with broader modification and versatility.^[46]^ Findings have developed to functionalize MOFs, increasing their diversity and scalability. Researchers have even begun to develop MOFs that extend from a two‐dimensional to a three‐dimensional spatial layout, all while maintaining their permanent porosity.[^47^, ^48^]

### 2.1 Synthesis of MOF structure

MOFs, also referred to as PCPs, are crystalline porous materials that contain infinite extended frameworks. These frameworks are constructed from secondary building blocks that consist of metal cations or clusters and polydentate organic ligands with various coordination modes. A wide spectrum of metal cations and linkers can be utilized during their potential post‐synthesis alterations.^[49]^ Because of their modular character, the synthesis process of MOF is immersed in both inorganic and organic chemistry, allowing multiple components in their structure with varying properties and functionalities (Table 1). The versatile nature of MOFs allows for the easy customization of the individual features of the MOFs. The diversity of MOFs can be further enriched by utilizing various amounts and types of organic ligands, each of which can have different lengths, shapes, and functional groups. The organic ligands can be of several different forms, such as linear, square, and triangular molecules, each of which has unique characteristics. Additionally, various types of metal ions can be utilized within the MOFs, such as divalent, trivalent, and tetravalent metal ions. The structural properties become more significant when the selection process of metal ions and organic ligands is precisely optimized for advanced and specific applications. MOF synthesis needs energy to break or establish ionic bonds because they comprise both organic binders and metal oxides.^[65]^ The primary objective is to preserve the integrity of the organic linkages while forming novel inorganic building blocks. Since there are various types of energy‐generating devices available, any device that can generate and control the amount of heat necessary for the synthesis process can be used for this step. The energy source and method of synthesis are used to determine the final MOF's characteristics. Several methods can be used to synthesize MOFs, including hydrothermal/solvothermal, ultrasonic (sonochemical), electrochemical, mechanochemical, microwave‐assisted, diffusion‐based, and evaporation‐based methods. Each of these methods exposes the reacting components to slightly different reaction conditions, which can impact the properties of the resulting MOF. A comparison of each of these synthesis methods is presented in Table 2. Among these approaches is the selection of synthesis conditions for the MOFs. Factors such as temperature, solvent, pH, reaction time, and metal‐to‐ligand ratio can all influence the formation of the secondary building units (SBUs) required to form the MOFs altogether.^[68]^ For example, the solvothermal method can easily provide favorable conditions for the formation of thermodynamically stable and highly crystalline frameworks having a three‐dimensional framework structure and possessing classical structures such as face‐centered cubic (UiO‐5 and MOF‐66), primitive cubic, and diamond networks through the slow self‐assembly of SBUs under high‐temperature and high‐pressure condition.[^69^, ^84^, ^85^] On the other hand, microwave‐assisted and mechanochemical synthesis methods are typically influenced by controlled kinetics, resulting in faster nucleation rates with the formation of metastable or defect‐rich phases, and even low‐dimensional structures (e.g., 1D chains or 2D layers), which are frequently classified as less common or distorted topologies. Synthesis techniques are extremely dependent on several reaction factors such as temperature, solvent type, pH, time, and metal and ligand ratio for SBU formation, laminar coordination geometry, and structural network interpenetration, which are responsible for distinguishing the class of MOF. As a result, careful selection and control of various factors are crucial not only for determining physical properties but also for targeting certain structural classes and topological families of MOF.^[70]^

**TABLE 1:** Various prepared MOFs with their synthesis method, composition, use, therapeutic compounds, and advantages.

Synthesis Process	MOF	Composition	Therapeutic compound	Application	Route of administration	Benifites	Ref.
Hydrothermal method	UiO‐66	Zr^4+^ + terephthalic acid	Ibuprofen	Drug delivery	Oral/injectable	High stability/pH‐dependent controlled release	[50]
	MIL‐100 (Fe)	Fe^3+^ + BTC	Dox	Cancer therapy	Injectable	High drug entrapment efficiency	[51]
	MIL‐101(Cr)	Cr^3+^ + BDC	Cisplatin	Chemotherapy	Injectable	Sustained release, high drug encapsulation	[52]
	ZIF‐8	Zn^2+^ + 2‐meyhylimidazole	5‐FU	pH‐responsive drug delivery	Injectable	pH‐responsive drug release at tumor environment	[53]
Mechanochemical	MIL‐100(Fe)	Fe^3+^ + BTC	5‐FU, aspirin	Anticancer drug encapsulation	Injectable/oral	Eco‐friendly and solvent‐free synthesis	[54]
	ZIF‐8	Zn^2+^ + Im	Vancomycin	Antibacterial effect	Topical	Green synthesis; scalable	
Electrochemical	Cu‐BTC MOF	Cu^2+^ + BTC	Cu^2+^ ions	Antibacterial/Biosensing	Topical	Tunable size	[55]
	Zn‐MOF thin films	Zn^2+^ + BDC	—	Diogenetic/Biosensing	Implantable sensor	High sensitivity/uniform film	[56]
Ambient temperature spinning	ZIF‐8	Zn^2+^ + Im	siRNA/DNA	Gene delivery	Injectable	Easy synthesis	[57]
	HKUST‐1	Cu^2+^ + BTC	Cu^2+^ release	Antimicrobial efficacy	Topical	Low energy is required during synthesis	[58]
Electrospinning	MIL‐101@PCL	Fe‐MOF + polycaprolactone	Growth factor	Tissue engineering scaffold	Implantable	ECM mimicking fibers; sustained release	[59]
	ZIF‐8@PAN nanofibers	Zn‐MOF + PAN	Zn^2+^ ions	Wound healing	Topical	Antibacterial, breathable mats	[60]
Carbonization	MOF‐derived porous carbon MOF‐derived porous carbon	Zn MOF‐carbon	Heat generation	Photothermal cancer therapy	Injectable	High stability and photothermal efficiency	[61]
	Fe‐MOF derived carbon	Fe‐C	ROS generate	Antibacterial therapy	Topical and injectable	Multimodal therapy with enhanced stability	[62]
Modified MOFs/Hybrid	HA@ZIF‐8	Hyaluronic acid + Zn^2+^ + Im	DOX	Targeted delivery	Injectable	CD44 targeting; tumor selectivity	[63]
	Chitosan‐based MOF	Zn^2+^ + Chitosan	Curcumin	Antimicrobial and wound healing	Topical	Improved biocompatibility	[64]

**TABLE 2:** Comparative analysis of MOF synthesis strategies: preparation conditions, structural consequences, performance characteristics, and scalability.

Synthesis method	Typical preparation conditions	Major advantages	Major limitations	Impact on MOF structure and properties	Implications for performance and biomedical applications	Scalability/translational considerations	References
Slow evaporation	Metal precursor and organic linker are dissolved in a suitable solvent and allowed to evaporate slowly, generally at room temperature; crystallization occurs as the solution gradually reaches supersaturation.	Simple equipment; room‐temperature operation; minimal energy input; suitable for controlled crystal growth and single‐crystal formation.	Very slow; strongly dependent on solvent volatility, concentration and ambient conditions; relatively low throughput; difficult to control nucleation uniformly at large scale.	Slow supersaturation generally favors controlled nucleation and growth, producing relatively well‐developed and highly crystalline particles/single crystals; crystal size can become relatively large.	High‐quality crystals are valuable for structural elucidation and structure–property investigations; however, large crystals and long synthesis times are generally less advantageous for nanoparticle‐based drug delivery.	Low scalability for high‐throughput production because of prolonged crystallization and dependence on evaporation kinetics; more suitable for discovery and structural studies than manufacturing.	[6, 65, 66]
Diffusion (liquid/gas/gel diffusion)	Reactants are placed in separate layers or compartments and allowed to diffuse gradually; gas‐phase, liquid‐phase and gel‐assisted diffusion can be used.	Mild conditions; low external energy requirement; slow reagent mixing allows controlled nucleation; often produces high‐quality single crystals.	Time‐consuming; low productivity; requires careful control of concentration, solvent density and diffusion rate; difficult to reproduce uniformly at large scale.	Slow diffusion reduces instantaneous supersaturation and can favor controlled nucleation, crystal growth and high crystallinity; useful for controlling crystal morphology and topology.	Particularly valuable for obtaining crystals suitable for structural characterization and understanding structure–function relationships; less suitable for rapid nanoparticle production.	Low scalability because of long reaction times, complex diffusion geometry and low throughput. Specialized counter‐diffusion approaches can, however, provide better control for MOF membrane fabrication.	[6, 65, 66, 67]
Hydrothermal/solvothermal	Metal salts and organic linkers are reacted in sealed vessels, commonly at elevated temperature and autogenous pressure; aqueous or organic solvents are used. Typical conditions vary widely with the MOF system.	Highly versatile; excellent control over crystallization; capable of producing highly crystalline and structurally robust MOFs; applicable to a broad range of metal/linker combinations.	High temperature/pressure; long reaction times; use of organic solvents such as DMF may create environmental and downstream‐processing concerns; batch processing can limit throughput.	Slow self‐assembly can favor thermodynamically stable phases, high crystallinity and well‐defined porous structures. Temperature, pH, concentration, modulator and metal/linker ratio strongly affect nucleation, crystal size, defects and topology.	High crystallinity and tunable pore architecture can provide reproducible loading and release characteristics, making the method important for MOF‐based drug delivery and other biomedical platforms.	Moderate scalability, but conventional batch reactors, solvent handling, energy demand and heat/mass‐transfer limitations remain challenges. Continuous‐flow and greener‐solvent adaptations improve scale‐up potential.	[67, 68, 69, 70, 71, 72]
Sonochemical/ultrasound‐assisted	Metal precursors and linkers are subjected to ultrasonic irradiation, commonly through acoustic cavitation; reaction conditions can be conducted at relatively low bulk temperatures and short times.	Rapid nucleation; short reaction time; relatively simple operation; can reduce particle size; useful for nanostructured MOFs.	Localized high temperature/pressure from cavitation can be difficult to control; scale‐up of uniform acoustic energy distribution is challenging; equipment‐dependent.	Cavitation accelerates nucleation and can produce smaller crystallites and modified morphologies. Reaction power, frequency and time can influence particle size, crystallinity and defect density.	Smaller particles and tunable morphology can improve dispersion, surface accessibility and cellular uptake, potentially benefiting nanoscale drug‐delivery systems.	Moderate scalability, but reactor geometry and homogeneous ultrasound distribution become important barriers at larger volumes.	[72, 73, 74]
Microwave‐assisted	Precursors are subjected to microwave irradiation, commonly in polar solvents, producing rapid volumetric heating; reaction times can range from minutes to substantially shorter periods than conventional heating.	Very rapid synthesis; efficient heating; reduced reaction time; often high yield; good control over nucleation and particle size; potentially lower energy consumption.	Requires specialized equipment; microwave penetration and heat distribution can become non‐uniform at larger scale; optimization is MOF‐specific.	Rapid and homogeneous heating can increase nucleation rates and produce smaller, more uniform particles; changes in temperature and irradiation power can alter crystallinity, morphology and defect concentration.	Rapid preparation of nanoscale MOFs with controlled particle size can be advantageous for drug/gene delivery and theranostic applications.	Moderate‐to‐high potential, particularly with continuous‐flow microwave reactors; however, scale‐up requires careful control of electromagnetic field distribution and heat transfer.	[72, 75]
Mechanochemical	Metal precursors and linkers are reacted by mechanical grinding, commonly in ball mills; liquid‐assisted grinding (LAG) may use small quantities of solvent.	Minimal or no bulk solvent; short reaction times; reduced waste; potentially lower environmental burden; inexpensive precursors can be used; compatible with solvent‐free processing.	Difficult control of temperature and mixing; particle morphology can be less uniform; mechanical energy input must be optimized; some systems may show reduced crystallinity or defects.	Mechanical energy accelerates bond formation and nucleation. Depending on milling conditions, products can exhibit high defect densities, altered crystallinity, smaller particles and different polymorphic/topological outcomes compared with solution synthesis.	Solvent‐minimized synthesis is attractive for biomedical materials where residual solvent is a concern; defect engineering may also provide additional active sites and altered degradation behavior.	High potential for scale‐up using industrial milling technologies, although uniform energy transfer, heat generation, milling‐media contamination and reproducibility require control.	[72, 76, 77, 78, 79]
Electrochemical	Metal ions are generated or released electrochemically from an electrode in the presence of organic ligands and an electrolyte; voltage/current, electrolyte composition and solvent influence crystallization.	Mild reaction conditions; relatively short synthesis times; reduced requirement for metal salts; good control of metal‐ion generation; potentially continuous operation.	Requires conductive electrodes and electrical control; electrolyte contamination may occur; electrode passivation and scale‐up of current distribution can be problematic.	Controlled metal‐ion release can influence nucleation and crystal growth, allowing control over particle size, morphology and crystallinity.	Can facilitate preparation of coatings and films and may enable controlled MOF deposition for biosensors, antimicrobial surfaces and biomedical devices.	High potential for continuous/large‐scale production, provided current density, electrode area, mass transfer and energy consumption can be controlled.	[68, 69, 72]
Room‐temperature stirring/precipitation	Metal salts and organic linkers are mixed and stirred under ambient or near‐ambient conditions for a defined period; water or relatively benign solvents can be used.	Simple; low energy requirement; inexpensive equipment; mild conditions; potentially suitable for sensitive functional groups and biomolecules.	May produce multiphase or poorly crystalline materials; nucleation and particle‐size control can be difficult; reaction outcome is highly dependent on precursor concentration and mixing.	Rapid mixing can increase nucleation density and generate small particles, but uncontrolled nucleation may result in broad size distributions, defects or amorphous phases.	Particularly attractive for biomolecule‐loaded MOFs and biomedical systems where high temperatures may degrade therapeutic cargo.	High potential because of mild conditions and simple equipment; continuous mixing/flow processing could improve reproducibility and throughput.	[72, 80]
Spray drying/aerosol‐assisted synthesis	Precursor solutions are atomized into droplets, followed by controlled solvent evaporation and crystallization/particle formation during drying.	Rapid processing; continuous operation; good potential for high throughput; can produce particles with controlled size and morphology; readily integrated with downstream powder processing.	Requires specialized equipment; rapid solvent removal may generate defects or incomplete crystallization; particle aggregation and morphology depend strongly on drying conditions.	Rapid concentration within droplets can promote nucleation and produce fine particles; droplet size, drying rate and precursor concentration influence crystallinity, porosity and morphology.	Particularly relevant for nanoparticle and powder production where particle size and dispersibility are important for delivery systems.	High scalability potential because spray drying is inherently amenable to continuous and industrial processing.	[66, 72]
Continuous‐flow/microfluidic synthesis	Metal and linker solutions are continuously mixed and reacted under controlled residence time, temperature and flow conditions; microreactors or continuous‐flow reactors may be used.	Excellent heat/mass transfer; reproducible reaction conditions; precise control of nucleation; continuous production; potentially reduced batch‐to‐batch variability.	Reactor fouling/blockage; optimization can be complex; downstream separation and scale‐out rather than simple scale‐up may be necessary.	Controlled mixing and residence time can produce relatively uniform particle sizes and crystallinity; nucleation and growth can be spatially and temporally controlled.	High reproducibility is attractive for biomedical MOFs, where particle size, loading and surface properties must be tightly controlled for biological performance.	Very high translational potential through continuous manufacturing and scale‐out; particularly attractive for reproducible production of nanoscale MOFs.	[66, 72, 81]
Carbonization/MOF‐derived materials	MOFs are thermally treated by calcination or pyrolysis under controlled atmosphere and temperature to transform the framework into porous carbon/metal‐containing derivatives.	Generates thermally stable porous materials; tunable composition; high surface area; can produce metal/metal oxide–carbon composites.	High energy demand; loss of original MOF framework; morphology and pore structure may collapse or transform; not appropriate when preservation of the parent MOF is required.	Thermal treatment dramatically changes the framework, producing porous carbon, metal nanoparticles, oxides or composite structures; surface chemistry, pore size and defect density depend strongly on temperature and atmosphere.	Useful for photothermal, catalytic, electrochemical and antimicrobial applications, but the resulting materials should not be considered equivalent to the parent MOF for drug‐delivery purposes.	Moderate scalability; thermal energy requirements and control of atmosphere/temperature are important manufacturing considerations.	[82, 83]

### 2.2 Hydrothermal/solvothermal method

The hydrothermal technique includes a number of methods for crystallizing materials from aqueous solutions at higher temperature and pressure. In the presence of a solvent, this reaction takes place, usually under near‐supercritical or supercritical conditions. MOFs are typically synthesized by mixing solutions of metal ion salts with organic ligands in specific solvents, which are then heated to very high temperatures (over 100°C) for a particular time. This extreme temperature helps to create a crystal yield within an experimentally feasible time frame. During this reaction, several factors like temperature, pH, and chemical compositions of reactive compounds are variable key parameters to maintain the integrity of this. Molecular templates or surfactants are also frequently utilized to develop nanostructures. The addition of metal ions and organic linkers can modify the pore size and volume of the MOFs. The hydrothermal approach has been used to create MOFs of various sizes, shapes, and crystal architectures. Even though thousands of MOFs have already been created, various criteria, such as surface charge, size, form, toxicity, and stability, must be carefully assessed before they are used in bio‐medical applications. All of these characteristics are linked; they frequently influence one another.[^71^, ^86^, ^87^]

### 2.3 Ultrasound (sonochemical) method

Ultrasound irradiation is a novel approach that has gained huge attention because of many in‐process benefits, which include ease of operation at favorable temperatures, selective product development, and a rapid reaction time. The usage of ultrasound in chemical reactions has increased in recent studies as a result of extensive investigation. Chemical processes are accelerated using strong ultrasonic pulses (20 kHz–1 MHz). Acoustic cavitation, the formation, expansion, and contraction of bubbles, influences sonochemical reactions in liquids. This causes intense heating, high pressures, and a short half‐life of bubbles, which have a significant impact on the reaction ambiance.^[73]^ In the presence of ultrasounds, many findings have demonstrated that MOF‐based porous materials can be efficiently synthesized, purified, and changed for their applicable purposes. Ultrasound has been shown to be an essential tool in MOF research, helping to minimize the particle sizes under more conditions than standard procedures that need high temperatures and extended reaction periods.^[74]^

### 2.4 Microwave approach

The microwave method involves heating the reaction mixture with microwave radiation, which has been shown to enhance the rate of crystallization of MOFs compared to other heating processes. The microwaves increase the rotation of certain molecules within the reaction mixture, which increases the production of heat caused by the collisions of those molecules. The increased production of heat within the reaction mixture expedites the reaction process overall. Microwave‐assisted synthesis has various advantages over hydrothermal synthesis, including faster reaction kinetics, phase purity, higher yields, and increased dependability and reproducibility.^[75]^

### 2.5 Mechanochemical approach

Mechanochemical reactions occur when solid reactant compounds usually absorb mechanical energy directly during the milling process. Friction and impact between the balls and reactants in ball milling cause chemical reactions.^[76]^ In order to initiate a reaction, the balls must have enough energy to break the chemical bonds within the metal oxide particles; otherwise, only elastic deformation will occur. High‐energy grinding creates the stress in the metal oxides that leads to the chemical bond breaking and the creation of reactive radicals. These created radicals allow the metal oxide particles to react with one another to form the desired MOFs. Mechanochemical synthesis involves the grinding of solid precursors in a ball mill in the absence of solvents. This method allows for the synthesis of MOFs with metal sources that are intractable in solvents. For example, metal oxides are insoluble in solvents and can be used instead of metal salts as an alternative to synthesis methods that employ solvents, rendering the synthesis process safer and more ecologically friendly. Another mechano‐chemical technique is Liquid‐Assisted Grinding (LAG), which employs a solvent to improve metal ion and organic bond mobility, hence increasing coordination bond formation.^[77]^

Ion and Liquid‐Assisted Grinding is a type of grinding that includes both salt and liquid in the milling process. These additives not only help to dissolve solid reagents but also increase the reactivity of the substrate, increasing the milling efficiency by resulting in a more uniform reaction mixture.[^76^, ^77^, ^78^] MOF synthesis must be carried out with an emphasis on economic and ecological factors. As a result, using easily available and reasonably priced precursors such as metal oxides is particularly desirable. By grinding nanosized ZnO with 2‐methylimidazole for 96 h, Taheri et al. discovered a dry method to produce ZIF‐8, with an SSA of 1480 m^2^ g^−1^, which is higher than the typical value of around 1200 m^2^ g^−1^ for this class of MOFs.^[79]^

### 2.6 Electrochemical method

For the synthesis of MOFs, the electrochemical technique is a continuous reaction process driven by redox potential. This method dissolves the ligand molecule and the electrolyte in the reaction chamber, with metal ions serving as the anode. Metal ions from the anode do not deposit on the cathode in the presence of protic solvents. Compared to conventional solvothermal or microwave‐assisted techniques, this method has a number of advantages, including gentler reaction conditions, simpler crystallization, and faster reaction durations. Moreover, continuous MOF production is made possible by electrochemical synthesis. Voltage, electrode current load, solution composition, electrolyte properties, and solvent selection all have a big impact on the ultimate structure of the MOFs.^[88]^

### 2.7 Ambient temperature stirring technique

The stirring method involves combining the metal precursors, the organic linkers, and the solvent. Then, the mixture is stirred at a specific temperature for a certain period of time.^[80]^ Zhao and his team utilized this method to create an ultrathin 2D ZIF‐67‐NS that effectively removes As(III) from water. ZIF‐67‐NS exhibited exceptional stability in the presence of alkaline conditions and had an improved adsorption capacity for As(III) due to its dimensional properties.^[89]^ Additionally, Tong and his research unit developed BZIF‐8‐containing biomolecular MOF, by agitating a mixture of Zn(CH_3_COO)_2_ 2H_2_O and 2‐methylimidazole at room temperature. This resulted in a multiphased BZIF‐8 structure with both crystalline and amorphous phases. The crystal electrostatic interactions between the precursors were identified as the growth process, and potential catalytic and pore transport capabilities were made possible by the amorphous phase's huge pore opening.^[90]^ In order to activate the metal compounds, Sui and his colleagues investigated the boronation of Ni‐Co MOFs at room temperature, followed by phosphating. The resulting material exhibited excellent stability for 87% of its original capacity after 5000 cycles of charging and discharging, and possessed remarkable electrochemical characteristics, such as a high capacitance of 1578 F/g.^[91]^

### 2.8 Electrospinning method

Electrospinning is a very effective method for MPFs synthesis into a nanofibrous system, which can fix several issues and improve many parameters such as stability, targeted delivery, and functional interaction with ligands and subjected compounds. In this process, a solution is subjected to high voltage, which contains polymeric compounds, producing nanoscale fibers into the MOF's structure. This chemical reaction promotes the formation of flexible, highly porous materials with a large surface area. This novel approach facilitates uniform distribution with a better composite system in several applications.^[92]^

### 2.9 Carbonization approach

This method involves extreme temperatures to develop highly stable MOFs. It occurs in the presence of some specific reactions called calcinations and pyrolysis, resulting in carbon nanomaterials with distinct characteristics.[^82^, ^93^] Controlling temperature and time during calcination can result in MOF‐derived carbon compounds with higher surface area and superior adsorptive properties. Modifying the composition of MOFs with additional organic compounds and metals can alter the properties of the resulting carbon.^[83]^ Collectively, these strategies demonstrate how thermal transformation of MOFs can be a powerful and versatile pathway to the development of future carbon materials with adaptable structures and functions that can be easily tailored to a wide range of environmental and catalytic requirements.

### 2.10 Classification of MOFs

MOFs are a distinct class of porous materials whose structures are different from those of purely organic or purely inorganic porous materials. MOFs consist of inorganic metal ions or metal clusters that are connected by multitopic organic ligands. In contrast, polymers are materials that consist of organic macromolecules, and COFs are porous crystalline materials that consist of organic components that are bonded to one another via covalent bonds.^[94]^ Zeolites and related molecular sieves, on the other hand, comprise inorganic materials, often based on aluminosilicate compositions. The incorporation of inorganic metal nodes and organic linkers into MOFs provides for the versatility of these materials in a variety of ways; the size of the pores within the MOFs, the chemical nature of the degrading MOFs, the charge of the MOFs, and how the MOFs interact with biological systems can each be engineered. Each of these characteristics is important in the context of potential biomedical applications of MOFs, such as in the delivery of drugs, and in MOFs that can provide both biologically active functions and diagnostic capabilities.^[95]^ Thus, the hybrid structure of MOFs provides an important structural foundation for engineering MOFs to provide a variety of biomedical functions. The relationship between structure and properties, designing a new framework with a desired function, and preparing MOFs for specific applications based on their classification. Additionally, these classifications are important for optimizing some useful purposes. Although many different applications have been investigated, the structural characteristics of MOFs cannot be sufficiently described by a single system.^[96]^

### 2.11 Pore‐size‐based classification

An important approach in classifying porous materials is according to the size of the pores within the materials. Porous materials are generally classified as having microporous (<2 nm), mesoporous (2–50 nm), or macroporous (>50 nm) structures. The size of the pores within MOFs can be engineered through the choice of the metallic nodes that form the MOFs, the organic molecules that link those metallic nodes to form the MOFs, and through structural or post‐synthetic chemistry strategies. MOFs can be synthesized to have micropores, mesopores, or macropores.^[97]^ For instance, microporous MOFs tend to have high specific surface areas, which allows for the adsorption of small molecules; however, the small pores may prevent the diffusion of larger molecules. Mesoporous MOFs have pore sizes that allow for the diffusion of larger therapeutic molecules, which can be advantageous in the delivery of larger drugs. Finally, macroporous MOFs have pores that allow for the transport of macromolecules; however, the large pores may reduce the adsorption of small drug molecules.^[98]^ Therefore, the pore size of MOFs is another important engineering parameter that must be balanced with other parameters, such as surface area, pore volume, stability of the MOF framework, and the interactions between the drug molecules and the MOF framework in order to achieve the desired loading and release of the drugs. Furthermore, MOFs that incorporate various classes of pores (micro, meso, and macropores) may enable various advantages in drug delivery, such as enhanced transport of the drugs and enhanced ability to provide controlled release of the drugs. Thus, this parameter is another that can be considered in the design of smart MOF nanoplatforms for targeted drug delivery.^[99]^

Pore size and pore aperture are two critical factors that determine the accessibility of molecules to the MOF's internal cavities. The pore size describes the size of the cavities within the MOFs, while the pore aperture or window size determines the entry and exit of molecules from these internal cavities. Therefore, a MOF may contain large cavities, but if the apertures into these cavities are smaller than the size of the therapeutic molecules, the molecules will not be able to enter the MOF's cavities. The size of the therapeutic molecules to be delivered is an essential parameter for the design of MOFs because MOFs need large apertures to allow for the therapeutic molecules to diffuse into the MOFs.^[100]^ However, large apertures will reduce the interactions between the MOF and the molecules. Thus, it is crucial to consider the size of both the cavity and aperture in the design of MOFs. The architecture of the MOF that is selected should be according to the dimensions and properties of the therapeutic molecules to be released from the MOF.^[101]^

### 2.12 Porosity engineering and structure–property relationships

Specific surface area and pore volume are two parameters that influence the loading of therapeutic molecules into MOFs. The SSA of MOFs, determined by the Brunauer‐Emmett‐Teller method, reflects the available internal and external surfaces for therapeutic molecule interactions. Higher SSA for MOFs provides more interaction sites for therapeutic molecules, which can lead to a higher loading capacity for these drugs. Pore volume represents the void space available within the MOF framework for relatively large molecules.^[80]^ High BET surface areas and pore volumes do not necessarily translate to high drug loading capacities for MOFs. The loading of molecules into MOFs can be influenced by several factors, such as pore accessibility, pore aperture, size and geometry of the molecules that are to be loaded into the MOF, and the strength of the host‐guest interactions between the MOF and the molecules. Thus, the parameters of interest include not only the surface area and pore volume of the MOFs but also their pore size and aperture.^[102]^ These parameters can be determined experimentally; however, the experimental measurement of the surface area and pore volume of MOFs can vary; they are influenced by the activation of the MOF framework, the removal of solvents from the MOF framework, the synthesis of the MOFs, and the method in which the measurements of these parameters are made.

Beyond the size of the pores within MOFs are other characteristics of these materials, such as their pore size distribution and pore connectivity. While the size of the pores within MOFs is often described for those MOFs, the pores within those materials can exhibit various dimensions within the MOF itself. Thus, each MOF can contain pores of various sizes, each with its own size distribution. Additionally, the connectivity of those pores within the MOF is also important in determining the movement of those molecular therapeutic agents within the MOFs. Consequently, each of these characteristics (along with pore size) influences the transport of those molecules within the MOF and can be engineered to provide for the efficient transport of the desired drug molecules from the MOF particles.^[103]^

Hierarchical and multimodal porosity is another strategy for enabling MOFs to contain pores of different sizes within the same structure. MOFs that contain micropores as well as mesopores and macropores (if any) can enable the MOFs to contain high levels of both adsorption sites (from the micropores) as well as facilitate the transport of the adsorbing molecules (from the larger pores).^[104]^ Thus, MOFs that incorporate such hierarchical porosity may enable the MOFs to overcome the transport limitations of MOFs that contain only micropores, while still retaining the high adsorption capabilities of MOFs that contain only micropores. In the context of the present nanoplatform, hierarchical porosity may be particularly beneficial in the need for the MOF to contain therapeutic molecules, allow for transport of those molecules, and release those molecules. The introduction of pores of different sizes, however, can have an impact upon the surface area of the MOF, the stability of the MOF, the mechanical integrity of the MOF, and the interaction between the MOF and the drugs that are to be delivered.^[105]^ Thus, the introduction of hierarchical porosity is best optimized according to the size of the therapeutic molecules that are to be delivered by the MOF nanoplatform.

Defect engineering provides another strategy for engineering the pore structure of MOFs. Defects in MOFs can be created by the intentional absence of organic linkers or metal clusters within the MOF structure; defects caused by the omission of organic linkers and metal clusters are among the most extensively studied. MOFs containing these structural defects often exhibit additional void space within the MOF structure, which can alter the accessible porosity of those MOFs. In some instances, the MOFs containing defects have exhibited the formation of additional (often larger) pores within the MOFs, enabling the diffusion of species that may not readily diffuse through microporous MOFs. Defects can be formed during the synthesis of the MOFs, or can be introduced through post‐synthetic treatments to the MOFs. In general, however, increasing the number of defects within a MOF can alter its structural integrity; increasing defect density can reduce the stability of the MOFs. Therefore, the concentration and spatial distribution of defects should be considered when designing MOFs in which enhanced pore accessibility and molecular transport are required.[^106^, ^107^]

Beyond the physical dimensions of the pores in MOFs, the chemistry of the walls of those pores also presents another important dimension of MOF porosity that can impact the properties of the encapsulated therapeutic molecules. The functional groups that can be incorporated into the MOFs during their synthesis or that can be introduced through post‐synthetic modification can impact the interaction between the MOFs and the therapeutic molecules that are loaded into the MOFs. These interactions can include hydrogen bonding, electrostatic interactions, π‐π interactions, van der Waals forces, and coordination interactions. Thus, each of these interaction forces can impact the loading of the MOFs with the therapeutic molecules, as well as the release of those loaded therapeutic molecules from the MOFs. For instance, modification of the surfaces of the MOFs to include the appropriate functional groups can enhance the interaction between the MOFs and the drug molecules, but weaker interactions may facilitate the release of the therapeutic molecules from the MOFs. Thus, another consideration in the engineering of MOFs for therapeutic delivery is the chemistry of the pores themselves. The chemistry of the pores can be engineered to interact with the therapeutic molecules in a way that provides an additional means of controlling the loading and release of those therapeutic molecules from the MOFs.[^108^, ^109^, ^110^]

Accessible porosity is another important consideration in the context of MOFs for drug delivery applications. Even if the theoretical dimensions of the pores within the MOF framework are sufficient to allow for the incorporation of the desired drug molecules, there may be issues with the accessibility of those pores. For instance, the pores may become blocked during the activation of the MOFs to remove any solvent molecules that were previously within the pores, the dimensions of the pores may be too small to permit the entry of the drug molecules, the MOFs may exhibit some flexibility that prevents the pores from readily becoming accessible to the drug molecules, or the drug molecules may interact with the MOF in a way that limits their accessibility. Each of these factors can potentially impact the amount of the drug that can be loaded into the MOFs, which is a critical consideration in relation to drug delivery. Therefore, characterization and optimization of MOFs for biomedical applications should distinguish between the theoretical pore structure and the experimentally accessible pore space.[^111^, ^112^]

Porosity‐dependent drug loading and release are two of the main features that must be considered in the development of MOFs for biomedical applications. The loading of drugs into MOFs is influenced by factors related to the pores of the MOFs (such as size, volume, and surface area), the structural dynamics of the MOFs, and the interaction between the drug and the MOF. MOFs with pores of an appropriate size for the molecular dimensions of the desired drug will allow the drug to become encapsulated by the MOF's pores. Too small pores will prevent the drugs from accessing the MOFs. MOFs with larger pores will allow the drugs to diffuse into the MOFs, but will not necessarily lead to increased loading of the drugs into the MOFs. The release of drugs from MOFs can occur through the diffusion of the drug from the MOFs' accessible pores, the degradation of the MOFs, or some combination of these two processes. Additionally, the flexibility of the MOF structure and changes in that MOF's structure can also impact the loading and release of drugs from those MOFs. Thus, the architecture of the MOF's pores should be matched to the size and properties of the drugs to be delivered, as well as the mechanism by which those drugs should be released from those MOFs.[^108^, ^112^]

The characterization and quantification of the porosity within MOFs is an essential step in determining the relationship between the MOF's structure and its function. One of the most common methods for determining the amount of surface area, pore volume, and distribution of pores within a solid is through the analysis of the physisorption of gases, such as nitrogen. The form of adsorption isotherms that are created from these analyses can reveal information regarding the MOF's textural characteristics, and mathematical models can be applied to those isotherms to determine the distribution of pores within the MOFs.^[113]^ For microporous materials, however, the gas that is adsorbed and the model that is applied to the adsorption isotherms are important; the assumptions of the BET model may not be appropriate for MOFs with such small pores. The use of gases like argon or carbon dioxide may provide additional information regarding MOFs that contain primarily narrow micropores or ultramicropores. Consequently, the reported values for the surface area and pore‐size distribution of MOFs should relate to the specific measurement conditions and methods employed to calculate those parameters.^[114]^ The use of such measurements and methods can aid in the comparison of MOFs with different proposed uses within the biomedicine field.

The modular construction of MOFs provides several routes for the tuning of the pore architecture of such frameworks. The metal nodes and organic linkers that make up the framework can each be tailored to alter the topology of the MOF, the dimensions of its pores, the aperture of those pores, and the chemistry that exists within the pores. For instance, the length, geometry, connectivity, and the functional groups that are present on the organic linkers can each be tailored to alter the size, shape, and chemical environment within the pores of MOFs. Furthermore, strategies like isoreticular expansion (where chemically similar yet longer linkers are incorporated into a MOF framework that is otherwise similar to a target MOF) can introduce additional dimensions into the MOFs. Additionally, coordination modulation and the creation of defects within the MOFs can similarly create additional void space within those frameworks. MOFs can also be synthesized with templates that lead to the formation of architectures that include multiple pores of different sizes. Finally, post‐synthetic approaches to MOFs, such as linker exchange or insertion reactions or covalent and coordinative modification reactions, can also reprogram the dimensions or functionality of the pores after the MOF framework has been synthesized. Thus, each of these approaches provides for the engineering of the porosity of MOFs across a range of length scales. These strategies can be employed based upon the size and properties of the molecules that are to be delivered by the MOFs, the stability of the MOFs, and the stability of the molecules within biological conditions.

The diversity of MOFs in terms of their pore architectures and textural properties is exemplified by several MOFs, such as MOF‐5, ZIF‐8, UiO‐66, MIL‐100, MIL‐101, and MOF‐177 (Table 3). Each of these MOFs contains different metal and organic components, leading to diverse pore sizes, surface areas, and volumes of MOFs, each of which can impart different properties onto small molecules that interact with the MOF and lead to their potential use in various biomedical applications.

**TABLE 3:** Representative MOFs with distinct pore characteristics and their relevance to biomedical applications.

MOF	Metal node	Organic linker	Pore characteristics	Specific surface area	Pore volume	Porosity class	Biomedical relevance	References
MOF‐5 (IRMOF‐1)	Zn_4_O cluster	1,4‐benzenedicarboxylate (BDC)	Large cavities; pore dimensions can be systematically tuned through linker extension	∼2900 m^2^ g^−1^ (Langmuir)	∼1.04 cm^3^ g^−1^	Microporous	Foundational platform demonstrating tunable pore architecture and functionality	[38]
IRMOF series	Zn_4_O cluster	Extended dicarboxylate linkers	Pore dimensions systematically varied from 3.8–28.8 Å across the reported series	—	—	Micro‐/larger‐pore regimes	Demonstrates rational control of pore dimensions through linker engineering	[115]
ZIF‐8	Zn^2+^	2‐methylimidazolate	11.6 Å pore diameter; 3.4 Å aperture	1947 m^2^ g^−1^	0.663 cm^3^ g^−1^	Microporous	Drug and biomolecule delivery; size‐selective molecular transport	[43]
UiO‐66	Zr_6_ cluster	BDC	∼7.5 and 12 Å cages; ∼6 Å aperture	∼1280 m^2^ g^−1^	∼0.46 cm^3^ g^−1^	Microporous	Drug delivery; chemically robust biomedical nanoplatform	[116]
MIL‐101(Cr)	Cr_3_ cluster	BDC	29 and 34 Å cages; ∼12 Å pentagonal and ∼14.5 × 16 Å hexagonal windows	∼5900 ± 300 m^2^ g^−1^ (Langmuir)	—	Mesoporous cages with microporous windows	Large‐molecule accommodation; drug delivery and imaging	[40]
MIL‐100(Fe)	Fe_3_ cluster	Benzene‐1,3,5‐tricarboxylate (BTC)	Multiscale pore system; reported pore diameter depends on sample/measurement	∼1604 m^2^ g^−1^	∼0.52 cm^3^ g^−1^	Micro/mesoporous	Drug loading and controlled delivery	[117]
MOF‐177	Zn_4_O cluster	1,3,5‐benzenetribenzoate (BTB)	Large interconnected cavities; reported pore diameter ∼10.6–12.7 Å	∼2970 m^2^ g^−1^ (BET)	∼1.11 cm^3^ g^−1^	Microporous	High‐capacity molecular adsorption; representative high‐surface‐area framework	[118]

### 2.13 Topology‐based classification

One of the most common methods for classifying MOFs is topological classification, which gives a clear geometry‐based view of the framework structure. The simplest form of MOFs consists of metal clusters (SBUs) as the nodes of the structure and organic linkers as the edges connecting the nodes. The various MOFs can be compared using this general structure. MOFs can be divided into different families based on their topology, including structures with motifs of the form dia, pcu, fcu, and many others with various unique structural features and geometries. Although topological analysis provides a strong framework for comprehending MOFs' spatial organization, it is frequently supplemented by additional classification techniques that emphasize other facets of MOF design.^[119]^

For example, there are dimensionality‐based classifications of MOFs according to the extension of connectivity in 1D, 2D, or 3D networks. Another classification method is based on the presence of cages within the MOFs. Additionally, MOFs can be classified according to the functional groups that are present within the MOFs. Finally, there are supramolecular classification methods for MOFs. Each of these classification methods can provide greater clarity to the structure of MOFs, which allows for their design according to the intended use of those materials.^[120]^

### 2.14 Classification based on dimensionality

Sometimes classification is based on structural dimensionality, which demonstrates the actual dimensionality of the framework. Zero‐dimensional (0D), one‐dimensional (1D), and three‐dimensional (3D) are the three principal categories to distinguish between. While 1D MOFs have chain‐like arrangements, 0D MOFs exist as distinct clusters or individual metal centres. 3D MOFs have vast networks with porous topologies. This significant classification describes valuable information about the relationships and spatial configurations of organic linkers and metal nodes in MOF.^[121]^

### 2.15 Cage‐based classification

*Cage‐based classification*: Some MOFs are classified based on the shape, size, and connectivity of their internal cavities. For instance, ZIFs have octahedral or cubic casings. On the other hand, structures like CPs and metal‐organic polyhedra often exhibit concave or hexagonal prismatic coordination cages. This unique classification is associated with precisely engineered pores that influence them into several potential applications for many purposes.^[96]^

### 2.16 Classification based on functional group

Another method for the classification of MOFs is to identify the existing functional groups found in their organic linkers. The presence of polar groups (‐NH_2_, ‐NO_2_, ‐OH, ‐CN, and ‐COOH), alkoxy groups (such as ethoxy, methoxy, and propyloxy), alkyl chains (e.g., ethyl, methyl, propyl, and longer hydrocarbons), and halogens (‐Cl, ‐F, ‐Br) have a substantial impact on MOFs' chemical, reactivity, and adsorption properties. This categorization emphasizes the chemical diversity of MOFs and their application potential, which is heavily influenced by the type of functional groups inside their structure.^[121]^

### 2.17 Supramolecular classification

The supramolecular method classifies MOFs based on their non‐covalent interactions and self‐assembly patterns. This technique considers important elements such as hydrogen bonding, π‐π interactions, host‐component dynamics, and coordination forces between metal nodes and entrapped molecules. Non‐covalent interactions strongly impact the stability, porosity, and usefulness of MOFs.^[122]^ Scientists can improve the molecular design and assembly procedures by organizing according to their interactions. This system often overlaps and combines several techniques that illustrate a better identification of its structure. Advancement of computational methods such as graph theory, machine learning, and data mining has enabled the establishment of automated MOFs classification, improving structural analysis and prediction.^[123]^ Nevertheless, no single classification has been established yet because of their complex structure.^[96]^

## 3 ENGINEERING SMART POROUS NANOPLATFORMS: DESIGN PRINCIPLES AND FUNCTIONAL EVOLUTION

In nanomedicine, MOFs are being transformed into smart porous nanoplatforms that can perform multiple functions. While many nanocarriers are created with the intention of maximizing the loading of the desired therapeutic agents, smart MOFs are engineered to perform a variety of functions in addition to delivering their payload; they can sense their environment, respond to stimuli, release their payload in response to those stimuli, and can even perform diagnostic and therapeutic functions simultaneously.^[124]^ Consequently, the concept of smartness goes beyond the structure of these MOFs to include their ability to interact with biological systems in specific manners. The intelligent behaviour of these MOFs emerges from their structural components. These MOFs can be tailored to include a variety of therapeutic agents within their pores. Additionally, the structure of these MOFs allows for the structural components to be controlled to respond to a variety of biological signals, such as pH levels, glutathione concentrations, ROS levels, ATP levels, hypoxia levels, and a variety of enzymes that are present in high concentrations within diseased tissues. These structural components allow the MOFs to release the encapsulated agents predominantly in the diseased tissues.^[125]^

Surface engineering further enhances the intelligence of MOF nanoplatforms. Functionalization with polyethylene glycol, hyaluronic acid, folic acid, antibodies, aptamers, cell membranes, and other biomimetic coatings improves the colloidal stability, circulation time, biodistribution, and targeting of MOF nanoparticles in biological systems. Another of the characteristics that defines the concept of smart porous MOFs is their multifunctionality. Most smart MOFs have been engineered to perform more than one function.^[126]^ For example, in addition to performing chemotherapy with incorporated chemotherapeutic drugs, MOFs can also deliver genes, perform photothermal, photodynamic, and chemodynamic therapy, and even perform multimodal imaging. Importantly, in addition to targeting diseased cells, smart MOFs also need to be engineered to ensure that they perform optimally in biological systems. Factors such as biodegradability, immunocompatibility, biosafety, and manufacturability must also be considered in the design and engineering of these systems.^[127]^ Thus, the intelligence and engineering of MOFs extend beyond their therapeutic efficacy to their applicability in biological systems. Therefore, throughout this review paper, the term “smart porous nanoplatforms” refers to MOFs that possess all the aforementioned features, making them suitable candidates for various biomedical applications of MOFs. Such an engineering perspective provides the foundation for the discussion of the various biomedical applications of MOFs in the subsequent review sections.

### 3.1 Key properties of MOFs in biomedical applications

This advanced composite system offers excellent benefits for various biomedical applications that include high therapeutic component entrapment efficiency with large surface area and controlled delivery and modification. Meaningful modification of structural chemistry and pore size allows targeted delivery with better therapeutic results.^[128]^ Their flexible features with structural versatility customize biocompatibility and biodegradability, making them more suitable for clinical needs. Surface modification with functionalized ligands and polymers improves colloidal stability, targeted therapy, and cellular uptake.^[129]^ Nanostructured MOFs also exhibit good multidistribution efficiency with enhanced permeable retention (EPR) effect in the tumor microenvironment.^[130]^ Several stimuli‐responsive MOFs are capable of releasing effective therapeutic compounds into a specific target in the presence of an acidic pH environment and redox potential conditions.^[131]^ In recent advancements of studies, Fe‐, Zn‐, and Zr‐based MOFs have shown lower toxicity and are easily biodegradable after use.^[132]^ Both therapeutic and imaging functions transform them into prominent theranostic platforms.^[133]^ Certain MOFs exhibit enzyme‐ or catalyst‐like activity for ROS‐assisted cancer therapy and biosensing.^[134]^ Moreover, this approach is applicable for transferring DNA, RNA, and several proteins, expanding the future research of gene therapy[^135^, ^136^] (Table 2). The application‐specific functional design strategies of MOFs in diverse fields of biomedicine are illustrated in Figure 1.

**FIGURE 1:**
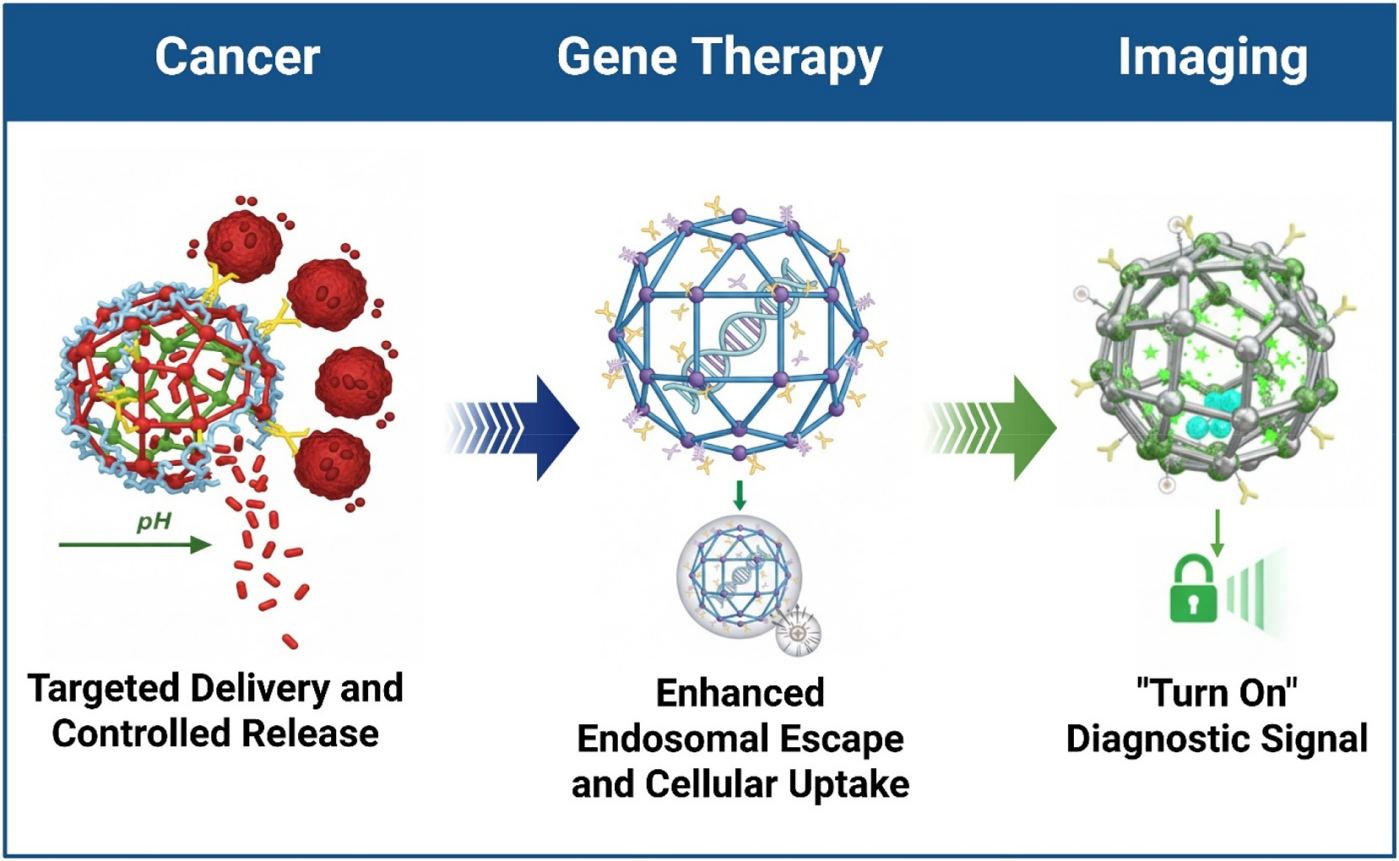
Application‐specific design principles of metal–organic frameworks (MOFs) in biomedicine. The functionalities of MOFs can be specifically designed for targeted drug delivery, immunotherapy, bioimaging, and phototherapy, such as photothermal therapy and photodynamic therapy. The modular structure of MOFs enables the design of MOFs to fulfill various therapeutic applications.

*Cancer therapy*: Cancer therapy is one of the most advanced applications of smart porous MOFs, with functionalities targeting tumors specifically, releasing drugs in response to stimuli from the tumors, and even killing cancer cells through the production of ROS.^[137]^ Zeng et al. developed MOFs that are functionalized with folic acid and are pH and redox responsive, allowing them to specifically target cancer cells, thus improving the targeted delivery of the system to the cancer cells. According to their findings, FA‐MOF can be a promising nanocarrier for Buf, enhancing anticancer efficiency. Administration of Buf at a dosage of 2 mg kg^−1^ prominently suppressed tumor growth in comparison to the control group. Additionally, all Buf formulations based on NPs showed stronger tumor inhibition than control Buf due to enhanced permeability and retention (EPR) effect. A. Treatment groups, on the other hand, showed a range of necrotic to apoptotic regions in the tumor tissues.^[19]^ In a recent study, Wang et al. developed an aptamer‐functionalized iron‐based MOF nanoplatform to create a synergistic effect when combining chemotherapy for simultaneous drug encapsulation and targeted drug release with chemodynamic therapy (CTD). This work developed an iron‐based MOF that encourages H_2_O_2_ to produce ROS for CTD in specific tissues. The engineered MOF was designed to improve cellular uptake and enhance therapeutic potential by lowering off‐target toxicity.^[138]^

Another study revealed the synthesis of MOF‐303 to target liver cancer cells. MOF‐303 is an aluminium‐based nanostructure with excellent stability and biocompatibility, enhanced cellular uptake, and a drug delivery system. Researchers visualized the distribution of high amounts of MOF‐303 within the cells through the endocytic pathway using X‐ray tomography. Results from this technique indicated the presence of MOF‐303 within the endosome and lysosome, where its acidic environment led to the release and degradation of the MOF‐303 payload. Such an imaging technique provides a distinct perspective on the trafficking of MOF‐303 and its unique properties, indicating that it can effectively target liver cancer cells while minimizing the effect on healthy cells.^[139]^

Furthermore, another study used MOF UIO‐66 functionalized with PRG (polyethylene glycol) and folic acid to achieve target‐specific delivery for breast cancer. Cisplatin and cyclophosphamide were co‐delivered within the MOF framework. The MOF exhibited significant encapsulation efficiency of cisplatin and cyclophosphamide (72% and 62%, respectively) within the FA‐PEG/(CIS + CYP)@UIO‐66 complex. The study revealed that MOF‐drug complexes upregulated markers of apoptosis (Bax, Caspase‐3, and 9) and downregulated invasion markers (MMP‐2 and 9) as compared to the control without the MOF‐drug complex. The use of such a pH‐responsive sophisticated technology allows for the release of the drug molecules at the tumor sites, enhancing the anticancer properties of the drugs while minimizing their toxicity to healthy cells.^[140]^ Furthermore, another discovery in this field includes the administration of doxorubicin (DOX) to target cases of triple‐negative breast cancer. DOX was encapsulated into a PEGylated MOF‐74 that was functionalized with transferrin to take advantage of the overexpression of the transferrin receptor on the cancer cells. As a result, the DOX MOF‐74 targeted the breast cancer cells and enhanced the uptake of DOX into the cancer cells, while preventing the binding of the drug to healthy cells and minimizing its toxicity to those healthy cells.^[20]^ In the paper by Kazemi et al., polydopamine was used to assist with the stability of the MOF, the dispersibility in water, and the ability to encapsulate DOX due to the π‐ and H‐bond interactions that occur between the PDA and the MOF; the acidic environment will allow for the medication to be released at the target location. Furthermore, because PDA can create ROS, it increases the ability of the nanoscale structure to escape from endosomes and lead to apoptosis in cancer cells. The increased ability of cancer cells to be killed while reducing the nonspecific killing of healthy cells indicates the effectiveness of the PDA to enhance the targeted delivery of chemotherapy to cancer cells. As such, Kazemi et al. have created an efficient and biocompatible delivery system that allows targeted and controlled chemotherapy to cancers.^[141]^ Similar to this study, Kazemi et al. also synthesized several other classes of MOFs at room temperature, including ZIF‐8, Fe BTC, Cu BTC, UIO‐66, and MOF‐5. Each of these MOFs was tested for its ability to target medication release in the acidic environment of the tumor while reducing its toxic effects on healthy cells. The targeted delivery of drugs like DNA, microtubules, folate receptors, Nuclear factor‐kappa B (NF‐κB), Bcl‐2, KRAS, and DHFR.^[142]^

MOF‐based nanoplatforms for cancer therapy are governed primarily by the sophistication of their engineering rather than their drug loading. Numerous studies have demonstrated that functionalization of the MOF nanoplatforms with targeting ligands and catalytic therapeutic agents increases the selectivity of the nanoplatforms for the tumors that they are engineered to target, while simultaneously reducing the toxicity of those nanoplatforms for the remainder of the body. Despite these research efforts, most studies have utilized only murine tumor models for testing of these nanoplatforms, and there are limited studies comparing different types of MOFs without utilizing different protocols for each MOF type. Future research should aim to compare the different types of MOFs, to study the long‐term effects of these nanoplatforms, and to determine methods for the scalable manufacturing of these nanoplatforms.

*Inflammation*: Beyond their use in cancer therapy, MOFs have also been developed for use in the treatment of inflammatory diseases, especially those that also include bacterial infections and issues with tissue regeneration. For instance, researchers have developed a zinc‐based MOF (Zn‐MOF) that exhibits properties that aid in wound healing, such as antibacterial, anti‐inflammatory, and tissue‐regenerative properties. Over time, the MOF released zinc ions that were effective in damaging bacterial membranes, preventing biofilm formation by bacteria, and promoting the growth of keratinocytes and fibrocytes. The MOF also helped to reduce hyperinflammation in the wound by reducing the release of cytokines such as TNF‐2 and IL‐6 that activate M2 cells. Zn‐MOF was studied for its ability to facilitate quick wound healing by inhibiting the growth of bacterial *Staphylococcus aureus* and *E. coli*, reducing oxidative stress, and promoting collagen deposition in the wound. Furthermore, because MOFs can provide a synergistic therapeutic effect by including features that regulate the immune system, kill bacteria, and restore tissue, they act as a beneficial component in wound healing treatment.^[143]^

A study was conducted on the use of Zn MOF functionalized hydrogels to modulate the anti‐inflammatory effects of ROS upon patients with atopic dermatitis (AD). The research discovered that the Zn^2+^ ions released from the MOF help to counteract the oxidative stress that exacerbates the disease symptoms. The suppression of ROS levels led to the reduction of the inflammatory response that follows, including the reduction of IL‐4, IL‐6, and TNF, which work to impede the healing of the skin barrier. The Zn‐MOF hydrogel successfully reduced the symptoms of AD in the mouse models by repairing the tight‐junctional proteins in the skin barrier and reducing TEWL. Thus, the Zn‐MOF hydrogel acts as an effective method of therapy for AD patients, working to both even out the oxidative environment in the skin as well as the inflammatory environment.^[144]^ Furthermore, another research study published in the journal used MOFs to treat cerebral ischemia reperfusion damage. The MOFs contained enzymes that sequestered superoxide dismutase and catalase, enzymes that break down ROS to prevent degradation of the cellular components, acting as biological ROS scavengers to reduce oxidative stress and the inflammatory response caused by NF‐κB activation. Furthermore, in vivo studies revealed that the MOFs decreased the number of dead neurons in the brains of the treated animals, as well as increased the perfusion of the cerebrum following the period of recovery from ischemia. Furthermore, the study indicated that targeted delivery of these MOFs to the brains of stroke patients is a viable form of therapy for those experiencing ischaemic stroke.^[145]^ Huang et al. investigated a photothermal hydrogel with bacteria‐capturing Bio‐MOF. The MOF components will bind to the bacteria, while the NIR light will break down the biofilm and kill the bacteria. This will provide antibacterial properties, as well as wound healing and anti‐inflammatory properties due to the extrusion of Zn^2+^ ions. Furthermore, healthy tissues at the wound site were found to be repairable. However, the restriction of permeability to deeply treated and overheated areas can cause irritation to the tissue.^[146]^ Furthermore, two natural compounds used to explore the antioxidant and anti‐inflammatory properties of the components were targeted by the MOF. For the treatment of inflammation, a more successful technique that reduces the number of cytokines present in the area was used. However, information on the potency of the toxicity of the treatment is yet to be explored and published in the paper by Huang et al.^[147]^

The findings of these studies demonstrate that smart MOFs have developed beyond the concept of simply delivering drugs to the affected area to a multifunctional capacity to modulate the immune system. The various engineering efforts for the MOFs have enhanced the anti‐inflammatory properties of the MOFs while reducing the negative effects of those MOFs. Despite these beneficial properties, the exact way in which the MOFs interact with the immune system and modulate it on a molecular level is not yet entirely understood. Future studies should investigate the MOFs' interaction with the immune system to further develop these therapies as potential clinical treatments for inflammatory diseases.

*Gene therapy*: The programmable pore architecture and the ability to engineer the surface chemistry of these MOFs render them as promising candidates for use as non‐viral gene delivery vectors. ZIF‐8, a zinc‐based MOF, has become a potent platform for non‐viral p53 gene therapy based upon its designated structural and chemical properties. The porous nature of ZIF‐8 allows for high surface area and pore volume properties that enable encapsulation of the p53 plasmid DNA molecules to prevent enzymatic degradation of the gene. Intracellular delivery of the p53 gene is possible in acidic pH environments typically found at the tumor site. The reinstated activity of the p53 gene leads to the inhibition of tumor growth by affecting the process of apoptosis, as well as by inhibiting the unnecessary growth of the tumor cells. Due to their biocompatibility, nanoscale size, and stimuli‐responsive nature, MOFs have introduced a safe and effective alternative to the viral vectors used in cancer gene therapy.^[136]^ Another study successfully developed a biomimetic nanostructure consisting of TPL and siHIF‐1α concurrently into Zr‐MOF (TPL/SIHIF‐1α@Zr‐MOF@CM). The NPs were successfully absorbed by the tumor cells. These NPs exhibit high biosafety and stability in vitro and in vivo. The researchers observed that the Kyse‐30 cells could efficiently absorb these NPs with a high drug loading rate. Under laser irradiation, the photosensitizer in these NPs produced ROS that killed the tumor cells. The oxidative damage at the tumor site was further increased by the suppression of TPL and HIF‐1α. The inherent characteristics of MOFs ensured effective drug delivery in vivo. Moreover, these NPs displayed a synergistic effect, leading to the enhanced anti‐tumor activity of the NPs in comparison with the control groups. Overall, this approach serves as a very effective alternative therapy for patients diagnosed with esophageal cancer.^[21]^ The work focused on creating efficient non‐viral vectors for gene delivery by functionalizing MOF NPs with polyethylenimine (PEI). The MOF has high porosity, a huge surface area, and an improved rate of adsorption of the nucleic acid. Additionally, PEI has a positive charge on its surface, strongly binds to nucleic acids, and allows for cellular uptake. The result of using PEI to functionalize the MOF NPs has resulted in a nanocarrier that has high entrapment efficiency of the nucleic acids, enzymatic degradation resistance, and a potent transfection rate. In vitro studies have demonstrated that these NPs have great potential for use in gene delivery due to their biocompatibility and improved cellular uptake and gene expression.^[148]^ In Huang et al.’s study, they developed a programmable DNAzymase based on rolling circle amplification (RCA) that was incorporated into a Cu/Zn bimetallic NMOF to create a platform named PZCT that was to be used in biology‐based bioorthogonal therapy using gene therapy. Zn^2+^ and Cu^2+^ were released in order to break the PZCT via the tumor microenvironment (TME). The RCA reaction that was triggered inside the cells as a result of miR‐21 ensured the targeted delivery of the DNAzyme to the tumor cells, and the amount of DNAzyme utilized was found to be sufficient in comparison to the traditional transfection of DNAzyme. The Zn^2+^ ions that were introduced into the PZCT system acted as a cofactor for the DNAzyme, which suppressed the development of the tumors by downregulating the expression of the EGR‐1 gene. Additionally, Cu^2+^ successfully guided chemodynamic therapy (CDT) to the tumor cells, leading to decreased viability of the tumor cells due to the conversion of H_2_O_2_ to the highly reactive OH radicals, as well as the scavenging of the intracellular GSH. Thus, both in vivo and in vitro models of tumor development were significantly suppressed by PZCT. Moreover, this study represents the first known use of the activation of RCA to generate therapeutic molecules for gene therapy, presenting a novel potential solution to the problem of the inefficiency of existing gene therapies for cancer treatment.^[149]^ Furthermore, compared to UiO‐66 and MIL‐88B, one group of researchers' ZIF‐based nanomaterial (zeolitic imidazolate framework‐8) MOF exhibited higher potential. IL8 was suppressed while IL10 remained unchanged, indicating that there was no immunotoxicity of gene@ZIF‐8. While ZIF‐8 that was washed with organic solvents has been used for the in vitro delivery of drugs, using water to wash the gene@ZIF‐8 instead was thought to favour the transport of the gene; however, further investigation is required in this area to determine the physical, chemical, and biochemical reasons why this may occur. Overall, though, this study sheds light on the important elements to consider in the effort of creating effective MOFs for gene delivery‐based treatments.^[136]^ As explained by Wu et al., one of the major limitations of using certain drugs to treat glioblastoma (GBM) is that the drugs exhibit low penetration rates through the blood‐brain barrier (BBB), preventing them from delivering the chemotherapeutic drugs, such as docetaxel (DTX), to the brain tumors effectively. They manufactured the construct RVG15‐PEG‐PEG@DTX‐ZIF‐8 by conjugating the 15 amino acid peptide Rhodevius germani (RVG15) to the surface of ZIF‐8 using the 15‐amino acid conjugate thiazide RVG15. This type of change allows for the targeted delivery of the nanosystem to the nicotinic acetylcholine receptors (nAChRs) that are expressed on GBM and brain endothelial cells, enabling the NPs to target the GBM tissue and cross the BBB. This type of NP was also shown to be highly biocompatible with the body, as it decreases the development of GBMs, reduces the metastasis of tumors formed by GBM, and increases the survival of mice with GBM tumors when used in animal models. Thus, this NP system presents a feasible method for utilizing MOFs to treat GBM.^[150]^

The goal of incorporating stimuli‐responsive degradation, biomimetic materials, or different therapeutic strategies into the MOFs has led to significantly improved transfection efficiencies compared to non‐viral vectors. Despite these improvements in MOF‐based gene therapy systems, most of these studies have been performed in preclinical models of disease. Consequently, the long‐term effects of MOF‐mediated gene therapy, as well as the safety of these MOF systems, are not yet fully understood. Future research should focus on determining the long‐term effects of MOF‐based gene therapy, incorporating gene‐editing strategies into MOF platforms, and testing these MOF systems in clinical models of disease. The multifunctional characteristics of MOFs are additionally related to anticancer mechanisms. Therefore, the following discussion will cover how MOFs can enable diagnosis and cancer treatment strategies to contribute to targeting tumors, delivering drugs to those tumors, modifying the tumor environment, or increasing the efficacy of cancer treatment for breast and gastrointestinal cancers.

*Bioimaging*: Beyond their ability to deliver genetic material to targeted cells, these smart MOFs can also act as multifunctional nanoplatforms that enable imaging of the targeted cells. For magnetic resonance bioimaging of 4T1 breast cancer cells, Jia et al. synthesized Gd/DTPA/MOF‐808/PANI (where Gd(III) is coupled with diethylenetriamine pentaacetate (DTPA) and polyaniline (PANI)). The occurrence of this synthesis resulted in an R1 value of 30.1 mM^−1^ s^−1^ (0.5 T). As the R1 value of this newly‐synthesized MOF‐based agent is far higher than the R1 value of the commercial contrast agent Magnevist, the potential of MOF‐based nano agents is seen as a promising topic for investigation in the area of diagnostic imaging.^[151]^ Furthermore, Zhu et al. prepared Fe‐DOX@Gd‐MOF‐ICG, where Gd^3+^ was used as the contrast agent for MRI to target cancer cells and deliver chemotherapeutic agents DOX and ICG to the target area following activation of the MOF core to release the agents. They utilized photoacoustic and photothermal imaging techniques on an imaging platform that contained the indocyanine green photosensitive chemical incorporated into the MOF. Incorporation of these imaging techniques yielded R1 and R2 relaxation values of 6.4 mM^−1^ s^−1^ and 81.9 mM^−1^ s^−1^, respectively. Pandit et al. synthesized a nanoparticle consisting of Fe_3_O_4_@ZIF8@ZIF67@folic acid by first preparing ZIF‐8@ZIF‐67 NPs with folic acid incorporated and Fe_3_O_4_ NPs, and coating the two types of NPs with the ZIF‐8@ZIF‐67 NPs with folic acid to target MCF‐7 breast cancer cells in MRI scans. Pandit et al.’s work displayed significantly higher R1 and R2 values at 2.042 and 85.86 mM^−1^ s^−1^, respectively. The findings of this study indicated an increased efficacy of iron oxide‐doped MOFs as MRI contrast agents, suggesting that the larger the amount of iron oxide incorporated into the MOFs, the higher the contrast of the MOFs in relation to the surrounding area.^[152]^ Pandit et al. have synthesized Fe_3_O_4_@ZIF8@ZIF67@folic acid coated by ZIF‐8@ZIF‐67 with folic acid and Fe_3_O_4_ NPs to specifically target MCF‐7 cells with MRI. The R1 and R2 values recorded by the authors are better than those of Zhu et al. (2.042 and 85.86 mM^−1^ s^−1^, respectively). The efficacy of iron oxide NPs within the MOFs was vastly increased by this development. Results also showed that larger amounts of iron oxide NPs within the MOFs indicated increased contrast within the MOFs.^[153]^ Lastly, researchers Bao et al. recently prepared a new contrast agent for computed tomography (CT) imaging with Hafnium (Hf^4+^) clusters and MnH_2_‐TCPP ligands to form Hf/Mn‐TCPP‐MOF (TCPP, 4,4,4,4‐(porphine‐5,10,15,20‐tetrayl)tetrakis as a contrast agent for CT imaging. The use of Hf (*Z* = 72) as the contrast agent for CT scans is based upon the element's enhanced ability to attenuate X‐rays. The results of the authors indicated that the CT value for the MOF was 70 HU, which is 1.7 times greater than the commercial contrast agent iohexol.^[154]^ Furthermore, Ma et al. synthesized NMOF545@Pt by combining HfCl_4_, Mn‐TCPP, and H_2_PtCl6. The resulting MOF had a CT value of 110 HU, which is better than the MOF created by Bao et al.^[155]^ You et al. also synthesized an MOF‐based treatment system that included CT, MRI, and FL. These synthesized MOFs used the 2‐aminoterephthalic acid (NH2‐BDC) ligand with Zr^4+^ modified with Au^+^ and Pt^+^. It was found that the longer the concentration of the Au on the MOF, the better the CT scan's resolution. The remarkable change in the MOFs' CT scan resolution was attributed to the incorporation of octahedral gold nanoshells (*Z* = 79), which significantly enhanced the reflecting properties of the MOF materials.^[156]^

For instance, other researchers have also synthesized MOFs with different properties. Wang et al. synthesized MOFs with near‐infrared fluorescence that could emit singlet oxygen when exposed to light of a specific wavelength by combining Zr^4+^ and H_2_L as the organic ligand. By injecting MOFs into the body and by analyzing these fluorescence images, the scientists aimed to locate the liver tumors that were present in the mice. These tumors on the mice were indicated by areas that exhibited high levels of red fluorescence for periods of up to 72 h after the MOF was injected into the mice. Furthermore, these MOFs can act as the carrier molecules for the fluorescent substances, such as the contrast agent indocyanine green, which is typically utilized in FL imaging.^[157]^ Furthermore, Yuan et al. created a fluorescent probe named DOX‐Gd‐TCPP‐MOF that can be used to diagnose the presence of 4T1 cells. The MOF that contains the DOX demonstrated promise as a treatment for the tumors, both in terms of its ability to kill the tumor cells and through its fluorescence properties in FL imaging. Furthermore, recent research has begun to publish the development of MOF‐based platforms that enable efficient and quick imaging of tumors in vivo.^[158]^

Current evidence demonstrates the evolution of engineered MOFs from imaging contrast agents to multifunctional agents that can be utilized for diagnosis, therapy, and monitoring of treatment efficacy. The rational incorporation of various magnetic, fluorescent, photoacoustic, and X‐ray attenuating components into MOFs has enabled enhanced imaging contrast and image‐guided therapy. However, challenges in biosafety, metal accumulation, and regulatory approval of MOFs still exist. Thus, future developments in this field should focus on biodegradable MOFs, standardized imaging metrics, and multimodal MOFs that combine imaging and therapeutic agents. An overview of representative MOF systems investigated across different therapeutic and diagnostic fields, along with their related payloads, stimuli, and clinical restrictions, is presented in Table 4.

**TABLE 4:** MOFs used in several studies with their payload, stimulus, the advantage, and limitation.

MOF type	Purpose	Payload	Stimulus	Advantage	Limitation	Reference
ZIF‐8 (Zn^2+^‐MOF)	Chemotherapy	Cisplatin, Doxorubicin	Acidic tumor microenvironment	Higher entrapment efficiency and controlled release	Toxicity issue: Presence of Zn^2+^ ions	[50]
MIL‐101(Fe^3+^ MOF)	Anticancer drug delivery	Paclitaxel, Sorafenib	Enzyme‐mediated degradation	Highly porous structure with excellent biocompatibility	Risk of excess iron accumulation	
UiO‐66/UiO‐67 (Ze^4+^‐MOF)	Gene delivery	pDNA, siRNA	pH‐triggered (endosomal)	High stability and enhanced nucleic acid protection	Reduced intracellular degradation rate	[159]
ZIF ‐90	Gene editing	CRISPR/Cas9	pH/ATP‐responsive degradation	Improved endosomal escape efficiency	Fewer in vivo studies	[160]
PCN‐224 (porphyrin‐containing MOF)	Photodynamic therapy	Porphyrins	Photo‐irradiation	Release of ROS	Restricted permeability of light in deeper tissue	[161]
Copper‐porphyrin‐based MOF	PTT (photothermal therapy)	Photothermal agents (PTAs)	NIR	High photothermal conversion efficiency	Risk of thermal damage to the surrounding tissue	[162]
MIL‐88B (Fe)	Immunotherapy	Antigens, adjuvants	Enzymatic degradation	Enhanced antigen‐presenting cell (APC) uptake and immune activation	Inflammation	[163]
ZIF‐8	Vaccine delivery	Antigens	Lysosomal acidic microenvironment	Immunoactivities with antigen protection	Long‐term safety	[164]
Gd‐based MOFs	MRI(Imaging)	Gd^3+^ contrast agents	Passive targeting	Enhanced proton relaxation	Toxicity occurred due to the uncontrolled release of Gd^3+^	[165]
Fe‐MOFs	Dual modal CT/MRI imaging	Ferrous clusters	Passive targeting	Bimodal imaging functionality	Accumulation of RES	[166]
Ag‐MOFs	Antimicrobial therapy (against bacteria)	Ag^+^ ions	Decomposition of MOFs	Potent antibacterial effect	Cytotoxicity	[167]
Drug‐dye co‐loaded hybrid MOFs	Theranostic	Drug and imaging probe conjugation	Light/pH	Diagnosis and therapy	Complex synthesis	[168]
ZIF8@polymer‐based MOFs	Stimuli‐responsive delivery	Therapeutic molecules	pH + redox	Controlled release	Challenges regarding large‐scale preparation	[169]

### 3.2 Design principles governing the clinical performance of smart MOF nanoplatforms

Unlike conventional nanocarriers, the therapeutic performance of smart porous MOFs is governed by several different parameters. The success of MOFs as drug delivery systems depends on the control of factors ranging from their structure to their degradation and manufacturing.^[184]^ Understanding the relationship between these parameters and the biological performance of MOFs is therefore one of the major objectives of current MOF engineering studies. One of the most important parameters governing the biological performance of MOFs is their size. MOFs with a diameter between 50 and 200 nm have been shown to exhibit enhanced properties related to their circulation in the blood, accumulation in tumors, and cellular internalization, whereas MOFs that are too large for these advantages are rapidly removed from the blood by the reticuloendothelial system (RES).^[185]^ Conversely, ultrasmall MOFs may be rapidly cleared from the bloodstream by the kidneys before they can reach the target tissues. Hence, controlling the size of MOF nanoparticles is essential for optimizing the drug delivery system efficiency.^[186]^

Another major determinant of the properties of MOF nanoparticles is their surface chemistry. The surface of MOF nanoparticles can be functionalized with various polymers (such as polyethylene glycol and hyaluronic acid), biopolymers (such as antibodies and aptamers), peptides, and even cell membranes in order to alter the way that the MOFs interact with biological fluids, cells, and targeting receptors.^[187]^ Finally, the structural composition of MOFs determines their degradation rates. MOFs can be designed with appropriate metals and linkers to form stable frameworks in the bloodstream. In addition, many new classes of MOFs are synthetized in such a way that they will only degrade in response to exposure to the pathological conditions that are typically present within target tissues (such as tumours), allowing the MOFs to remain stable and deliver their payloads only after reaching the targeted cells.^[188]^

Another emerging design principle is the integration of multiple therapeutic modalities within these MOFs. In addition to delivering chemotherapeutic drugs to cancer cells, MOFs can also be engineered to deliver genes, catalytic agents, photothermal agents, photodynamic agents, immunotherapy agents, and enable multimodal imaging. This allows for synergistic therapeutic effects as well as monitoring of the treatment.^[133]^ Finally, engineering strategies that improve the translation of these drugs to the clinic are being considered. In addition to efficacy, MOFs must also be engineered to meet the requirements regarding manufacturing, sterilization, storage, biosafety, biodegradability, and regulatory requirements. Thus, engineering smart porous MOFs will be guided by both engineering and translational considerations in the future.^[184]^

Overall, these engineering principles highlight the fact that the design of smart porous MOFs is not based upon individual properties of the materials, but instead in the interplay between the requirements of biology, medicine, and engineering. This provides a rational framework for understanding the topics that will be discussed in the following sections.

### 3.3 ADME of MOF involves biological distribution, degradation, accumulation, and clearance

Based on the engineering principles discussed above, the biological fate of MOFs after being introduced into the biobodies determines their ultimate biomedical performance. Regardless of the advanced engineering designs implemented into these MOFs, favourable absorption, degradation, and clearance from the body are required for them to be successfully translated into clinical applications. Thus, understanding the ADME characteristics of these engineered nanomaterials is an essential aspect of their design. The biological fate and lifecycle of MOFs, from synthesis to degradation, are represented in Figure 2. Such frameworks are formed by metals and CPs that are connected to organic linkers. The biodistribution of MOFs occurs through the dissociation of the metal‐ligand bond. The degradation of MOFs occurs due to various environmental factors such as pH, redox conditions, and the concentration of ions present in the body, which aid in the release of the metal ions and the ligand that encapsulates the therapeutic agent.^[99]^ The properties of the metal nodes that make up the MOFs also play a vital role in the degradation of MOFs. MOFs with softer and more labile linkers will degrade at a faster rate than those with stable Zn‐ or Fe‐based metal clusters. Furthermore, elucidating the degradation rate of MOFs and characterizing them is vital to understanding how to balance the therapeutic and biosafety effects of these MOFs.

**FIGURE 2:**
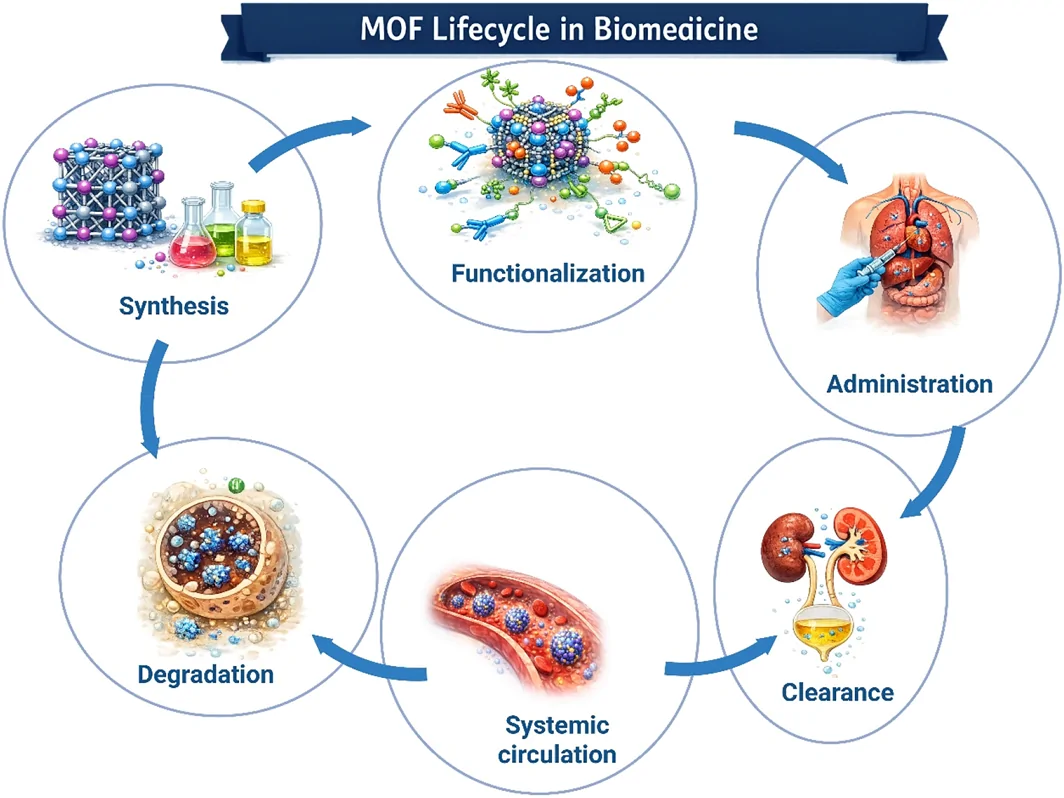
Lifecycle of metal–organic frameworks (MOFs). After being synthesized and functionally modified, MOFs are administered to the biological system. After entering the system, the MOFs interact with the target tissues, are degraded, and are excreted from the body through the kidneys or digestive system. Understanding the fate and elimination of MOFs from the body allows for their characterization and optimization for desired pharmacokinetic, biosafety, and therapeutic efficacy properties.

The biodistribution of MOFs within biological systems can vary greatly depending on the physicochemical and size characteristics of the MOFs. After intravenous administration, MOFs will bind to the RES in the bloodstream and will distribute to the liver, kidney, spleen, and lungs. The enrichment of MOFs within the RES does not indicate toxicity to the body but indicates the physiological pathways through which MOFs are cleared from the body.^[189]^ The use of surface engineering strategies, such as utilizing biocompatible or “stealth” coatings for MOFs, can reduce the amount of MOFs that are enriched within the RES, thus increasing the length of the circulation time of MOFs and the amount of MOFs that can reach their target cells. After reaching their target cells, MOFs can be cleared from the body through the renal and hepatobiliary elimination mechanisms. Small molecule fragments and compounds of low molecular weight, as well as free metal ions and hydrophilic fragments of MOFs, can be rapidly eliminated by our vital organ, the kidney.^[190]^ Molecules that are larger in molecular weight than those rapidly eliminated by the kidney can be cleared by hepatic cells. Biliary system clearance occurs for the waste products that are water‐soluble and eliminated in the feces. Therefore, size, the chemistry of the MOFs' surface, the rate at which MOFs degrade, and the formation of protein complexes with MOFs will impact the way in which MOFs are distributed within the body and cleared from the body over time, and hence their biosafety.

Finally, the time‐dependent fate determines the pharmacokinetics profile of MOFs from the time they enter the systemic circulation until their eventual clearance from the body, playing a critical role in the therapeutic potential and biosafety of these nano‐engineered frameworks. The rapid degradation of MOFs may result in the release of the encapsulated payload prior to the accumulation of MOFs at the target tissue. However, a slower rate of degradation or an increased stability of MOFs may lead to prolonged therapeutic effect at the expense of increased toxicity of the MOFs. Hence, understanding the in vivo fate of MOFs, including their distribution within specific organs, their degradation, and their clearance from the body, is critically important in understanding how they may be best utilized in biomedicine and other therapeutic disciplines.[^191^, ^192^, ^193^, ^194^, ^195^]

## 4 DETERMINANTS OF MOF SAFETY: INNATE IMMUNE ACTIVATION, CYTOKINE RESPONSES, IMMUNOGENICITY, AND SURFACE MODIFICATION STRATEGIES TO AVOID IMMUNE CLEARANCE

MOFs interact with the immune system, which is governed by their physicochemical properties. The innate immune system uses macrophages of the RES and the activation of the complement system to recognize the exogenous materials of MOFs and initiate a phagocytic response to remove those foreign substances. It can trigger variable cytokine responses, including the release of pro‐inflammatory mediators, like TNF‐α, IL‐6, and IL‐1β, which may affect efficacy and safety outcomes. Immunogenicity may be beneficial for vaccine applications. Immunologically inert MOFs often engage in foregoing immune camouflage and achieve prolonged circulation.^[196]^

Surface engineering techniques like PEGylation, stealth properties, biomimetic or zwitterionic functionalization are also being explored to minimize protein adsorption, complement activation, and immune rejection, making it the key interaction in immunology with a better translational and safety profile.[^50^, ^197^, ^198^, ^199^]

### 4.1 Clinical advancement and regulatory framework

The clinical advancement of MOFs in the field of biomedicine will require compliance with the standards of the various regulatory bodies. Regulatory authorities such as the US Food and Drug Administration (US‐FDA) and the European Medicines Agency will have to authorize MOFs for use in medicine based on analyses of their quality control, effectiveness, safety, biocompatibility, and dosing of these nanomaterials, including MOF‐based delivery systems.^[96]^

Evaluation of MOF‐based delivery systems as nanomedicine products requires physicochemical and biological evaluations according to established manufacturing guidelines. The physicochemical properties of MOF nanoparticles influence the long‐term safety profile of the nanomedicine. Toxicological evaluation is essential for MOF‐based nanomedicine products due to their inorganic metallic compositions. Regulatory guidelines require in vitro and in vivo toxicity studies to evaluate the safety of MOF‐based nanomedicine products and to rule out any potentially toxic effects of these NPs.[^51^, ^200^, ^201^, ^202^] The translational pathway of MOF‐based nanomedicines from the laboratory to clinic is depicted in Figure 3.

**FIGURE 3:**
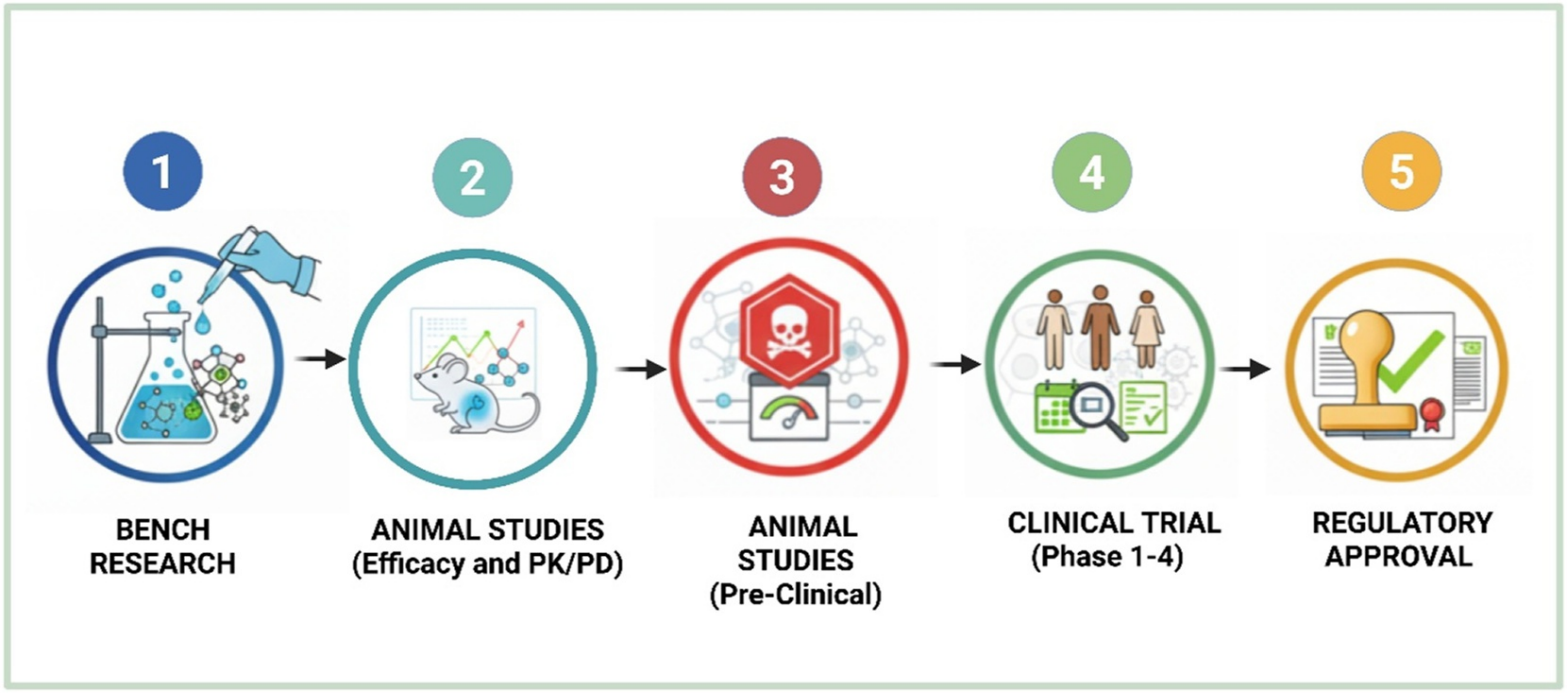
Clinical translation pathway of metal–organic framework (MOF)‐based nanomedicine. The process includes research, animal studies, toxicity studies, clinical trials (phases I‐IV), and regulatory approval for MOF‐based nanomedicines. The translational research process highlights the challenges and necessary studies for the advancement of MOF‐based nanomedicines into the clinical setting.

### 4.2 Scalable manufacturing and process reproducibility

The scalability and reproducibility of MOF synthesis processes within the manufacturing stage are the biggest challenges for MOF‐based drug delivery systems. The synthesis processes that are commonly used in MOF synthesis led to batch variability in the drug loading, size, and crystallinity of the synthesized MOFs. Manufacturing processes that meet the necessary regulatory requirements must be established to address these issues. Methods for overcoming the scalability and reproducibility challenges to MOF synthesis include utilizing continuous flow reactors, employing green solvent methods for MOF synthesis, and using microwave‐assisted synthesis methods. The quality by design approach and process analytical technologies are being used to ensure reproducible synthesis of MOFs. Additionally, the standardization of synthesis processes is key to the production of clinical‐grade MOFs.[^6^, ^203^, ^204^, ^205^]

## 5 ADHERENCE TO GMP AND MANUFACTURING REQUIREMENTS

The procedure must be followed accurately to prepare MOFs under good manufacturing practice (GMP). It will ensure the integrity of the MOFs, improve the purity of the MOFs, and ensure that they are traceable throughout the manufacturing process. Quality control, method validation, avoiding cross‐contamination, and proper documentation will be necessary. To maintain the aseptic nature of the pharmaceutical product, proper sterilization processes should be maintained.^[206]^ In order to determine the shelf life stability of the product, it is first necessary to perform testing under proper GMP conditions and then store the product under suitable conditions and humidity levels. Finally, in the case of the production of these medications on a large scale, the control of GMP conditions will be critical for the medications to be clinically translated from the laboratory preparations to the patients who will receive them.

### 5.1 Regulatory concern and safety considerations associated with specific metals (Zr, Fe, Cu, Hf)

The presence of metallic ions such as Hf, Cu, Zr, and Fe may enhance the chance of toxicity in the biological system. Regulatory agencies carefully evaluate the safety of metal‐based MOFs. Zirconium‐based MOFs such as UiO‐66 exhibit excellent chemical stability and less toxicity in biological systems, making them highly promising for future applications.[^207^, ^208^] MOFs based on ferrous ions, such as MIL‐101 and MIL‐88, show clinical potency in biological systems with established biological pathways due to the presence of iron in the biological system, which mainly reduces MOF toxicity and enhances the accumulation of MOFs in the biological system. This enhances regulatory acceptance.[^209^, ^210^] Copper (Cu) based MOFs are considered to have moderate regulatory concern due to the redox potential of copper to induce oxidative stress on biological systems at high concentrations.[^211^, ^212^] Furthermore, excessive amounts of copper in biological systems can lead to cytotoxicity and inflammatory responses in those systems.[^213^, ^214^]

Another one that has received considerable attention includes Hf ion‐based MOFs (Hf‐MOFs) that can be utilized in radiotherapy. Hf ions have a high atomic number and are found within a minimal range in our biological system. Therefore, ingesting this compound through the MOF‐based biomedicine system can present challenges and considerations regarding the utmost priority of regulatory considerations prior to clinical use.[^214^, ^215^, ^216^]

### 5.2 Current clinical trial

The potential biomedical applications of such a discovery have been supported by a number of studies, including patents (Table 5) and clinical trials.^[217]^ However, careful considerations must also be made regarding the need for more studies to be performed in the field of human studies to determine the safety and effectiveness of the treatments for inclusion in clinical trials for future pathways.^[96]^ The main goal of performing clinical trials is to test the safety and efficacy of the medical treatment in question, not just how non‐toxic the treatment is for patients. Clinical trials involve a lot of responsibility; clinical trials can only be performed after a thorough study of the treatment has been conducted. Even so, there is always some risk associated with clinical trials due to the fact that each patient's immune system may react negatively to the treatment in question, despite the number of legitimate and trustworthy studies that have been published regarding the treatment. Thus, the transition of a treatment from research to clinical trials can be a difficult and drawn‐out procedure. Despite the difficulties associated with clinical trials and the lengthy process of performing them, research in this area is crucial to the potential development of effective treatments for the immunosuppressive conditions described above.

**TABLE 5:** Summary of recent patents and applications related to metal–organic framework MOFs.

Patent/application	Patent	Purpose	Objective	Reference
MOFs deliver small molecules and biomacromolecules for cancer immunotherapy	WO2021237209A1	Cancer‐specific MOF‐based system	Modification and improvement of MOFs that bind therapeutic compounds for immunotherapy	[170]
Metal‐organic framework‐mediated macromolecule delivery.	WO2022103833A1	Biomolecules and therapeutic compounds delivery	MOFs designed for in vivo stability and controlled release of medicinal biomolecules	[171]
Metal organic framework materials with antibacterial properties	US11452967B2	Bioactive MOFs	For antibacterial function and several biological contexts	[172]
Metal organic framework polymer composites	US11007391B1	Composite MOF materials	MOFs combined with polymers are used in biological interfaces and therapeutic material scaffolds	[173]
Production of metal‐organic framework	US20200108369A1	Synthesis method of MOFs	Scalable production and reproducible nanostructure biomedical uses	[174]
Metal‐organic framework material and preparation method thereof	CN114044913B	Industrial scale preparation	Materials and methods of preparation	[175]
Polymer metal‐organic framework composites	US10561761B2	Polymer metal organic framework	Mechanically robust, stable composites for industrial and biomedical applications	[176]
Composites for transdermal delivery utilizing metal‐organic frameworks and nanocellulose	CN111526857B	MOF based on transdermal delivery	MOF nanocellulose composite system for transdermal delivery efficiency of therapeutic compound	[177]
Quercetin drug delivery system based on copper sulfide‐metal organic framework material	CN108524935B	Copper sulfide‐based MOF for quercetin drug delivery system	Controlled and efficient release of quercetin	[178]
Edible and biocompatible metal‐organic frameworks	EP2190662B1	Edible and biocompatible MOFs	Safe use of MOFs in biomedical and pharmaceutical applications	[179]
Solid‐state crystallization of metal‐organic frameworks within mesoporous materials, methods, and hybrid materials thereof	US12116286B2	Solid‐state method to develop crystallizing MOF in mesoporous materials	Enhanced adsorption, sensing, catalysis, and separation applications	[180]
Biopolymers and metal‐organic frameworks conjugated complexes and uses thereof	KR102264231B1	To develop a conjugated complex of biopolymer and MOFs	Enhanced application in drug delivery, sensing, and biomedical	[181]
Preparation method of nano metal particle/metal organic framework composite antibacterial cellulose fiber	CN108589266B	To develop a stable and effective composite system	Strong stability and antibacterial efficacy against *Staphylococcus aureus* and E. coli	[182]
Method for the preparation of metal‐organic frameworks	US9352489B2	Novel and industrial method of preparation	High quality and large‐scale preparation with overcoming many limitations	[183]

The clinical phase takes a lot of time, from a few years to many decades, to complete, even though the development of these materials is good. This extended timeline is the result of a number of different variables, including the length of the treatment process itself, the need for stringent ethical and regulatory permissions, and the necessity of obtaining volunteers from various countries and age groups.^[96]^ There have been extensive studies on MOFs that have led to the discovery of nearly 90,000 new structures of these materials.^[218]^ Only two of these MOFs have advanced to the clinical trials phase with human participants as detailed in Table 6: RiMO‐401, which is in phase I clinical trials (NCT06182579), and RiMO‐301, which is in phase II clinical trials (NCT0583872910).^[219]^ RiMO‐301 is an Hf‐based MOF that has shown promise in increasing the effectiveness of radiation and checkpoint blockade immunotherapy while avoiding toxic effects on the human body in animal models.^[220]^ However, numerous experts in the field have expressed concern as to why it has taken so long for these MOFs to reach the clinical phase with humans, citing some of the challenges to their development and clinical approval.

**TABLE 6:** Overview of clinical‐stage MOF‐based therapeutics.

Name and ID	Type	Phase	Sponsor	Application	Route of administration	Key outcome	Reference
RiMO‐301 (NCT05838729)	MOF based on hafnium (Hf‐MOF)	II	Coordination pharmaceuticals, Inc.	Treatment of head and neck cancer	Intratumoral injection	Improved immune checkpoint blockade and radiation efficacy in preclinical models; demonstrated no systemic toxicity and acceptable tolerance; progressed to phase II	[219, 220]
RiMO‐401(NCT06182579)	MOF based on hafnium (Hf‐MOF)	I		Solid tumor radiosensitizer	Intratumoral injection	First‐in‐human safety assessment; intended to increase the production of ROS during X‐ray irradiation; clinical data is currently being gathered	[219]

One issue that has been identified as a potential obstacle is biocompatibility. The potential toxicity of the MOFs based upon their composition is additionally complicated by the ability of the inorganic compounds to form a variety of different crystal structures. Each of the components of the MOFs must be evaluated separately to determine their potential toxicity, including their size, shape, and the formulation of the MOFs in relation to the body systems and components in question. Furthermore, the instability of MOFs in biological fluids like blood and serum can present obstacles to their translation into biological applications. The metal centres within MOFs are often labile, allowing for biological anions such as phosphate or biological molecules such as the protein albumin to strip the MOF of its metal centres, leading to the potential premature release of the encapsulated drug molecule, deterioration of the MOF, and subsequently, decreased efficacy of the MOF‐based drug delivery system.^[221]^

### 5.3 Software‐based screening for MOFs

Computer‐aided design involves using software to model MOFs in a way that accelerates their translation into clinical applications. Safety‐related design of MOFs can be performed prior to in vivo and in vitro testing. Furthermore, predictions regarding MOF stability and efficiency as a therapeutic agent can be made based upon the physiological conditions under which the MOFs are to be utilized, utilizing methods like density functional theory calculations to determine the strength of the metal‐ligand bonds within MOFs.^[222]^ These calculations are often implemented in programs such as VASP, CASTEP, and Gaussian. Additionally, molecular dynamics simulations can help to determine the interaction of MOFs with biological components and systems. MOFs can be simulated using software packages like GROMACS, LAMMPS, and AMBER. In order to optimize the drug‐loading and ‐release of MOFs, the use of GCMC simulations using software like RASPA and Materials Studio can be used to accurately predict how the MOFs will encapsulate and release the drugs.^[223]^ Furthermore, through the use of high‐throughput computational screening methods in conjunction with databases that contain various MOFs, such as CoRE MOF, MOF‐DB, and the Materials Project, the safety of those MOFs can be validated. Each of these CAD systems can greatly reduce the number of experiments that must be performed to test each MOF for drug delivery applications, and they are becoming more and more recognized as essential instruments in the effort to gain regulatory clearances and utilize MOF‐based nanomedicines in clinical settings.[^223^, ^224^]

## 6 TOXICITY

### 6.1 Cytotoxicity due to physicochemical properties

Generally, NPs are prepared by using different chemical compositions and polymers. In a similar way, each of these factors can impact the toxicology and harmful properties of MOFs. Therefore, in order to ascertain the toxicity of MOFs, it is essential to determine the unwanted effects of each component of the MOFs. The greater part of MOFs currently in use for drug delivery systems employ Fe and Zn, raising concerns regarding the toxicity of these metals on a short‐term basis. However, there are many unknown answers regarding the effects of these metals over the course of long‐term experiments on animal studies. The oxidation state of the metal center in these MOFs also has a substantial influence on the toxicity of the MOFs. Some organic linkers utilize the cytotoxic effect of MOFs as they contain the same metals as the MOFs, such as benzene‐1,4‐dicarboxylic acid and 2‐butenedioic acid, which have been used to create UiO‐64(Zr) and UiO‐66(Zr), respectively, to evaluate their harmful effects on J774A.1 cell. Sifaoui et al. demonstrated that these MOFs did not exhibit toxicity due to the biocompatibility of Zr(IV).^[225]^ The metal ions that are released from the MOFs within the biological system can hamper the cellular mechanisms within our biological system. Thus, it is important to consider the various toxic effects that can be created by MOFs that contain different metal ions but have the same organic linker. Kumar et al., for instance, have studied the various metal ions that are present within MOFs, finding that the most applicable metal ions include Co(II), Cu(II), Cd(II), Zn(II), Fe(II/III), and Ni(II).^[226]^ Furthermore, another team of researchers headed by Horcajada studied the impact of MIL‐100 MOFs that contained Fe, Cr, and Al metals on HepG2, Calu‐3, A549, and Hep3B cell lines. Their results indicated that MIL‐100 containing iron (Fe) exhibited significant cytotoxicity only within the Hep3B cell line.^[227]^ On the other hand, MIL‐100 containing chromium (Cr) and aluminum (Al) metals did not exhibit in vitro toxicity. Such findings indicate that there may be a correlation between the toxicity of the samples and the release of iron cations into the medium or the particles themselves.^[228]^ Besides the MILs, there were other MOFs under investigation with regard to evaluating the toxicity that their metal nodes can introduce into the body. The effect of ZIF‐8 and ZIF‐67 on hematology was compared, two materials that used the same linkers of 2‐methyl imidazole, yet utilized Zn and Co metal ions, respectively, as the coordination center. ZIF‐8 was found to be hemocompatible, yet ZIF‐67 was found to be hepatotoxic due to the membrane osmolarity dysfunction that results from the action of ROS. Thus, it was found that the difference in the metal ions that were used in the framework of the MOFs led to a significant difference in the safety of those MOFs.^[229]^ MOFs that contain metals that are toxic to the human body, such as Cd, Ar, Pb, and Cr, often contain effects that are critical to those metal ions yet harmful to the body due to their tendency to interact with biological molecules and create ROS. MOFs that contain natural biodegradable elements, such as zinc and iron, however, tend to have less harmful effects of those metals entering the human body and exerting their effects upon the system.^[210]^

### 6.2 Toxicity related to biological functions

Additionally, different parameters have been used to assess the toxicity of MOFs on living organisms, including changing the concentrations and in what way the MOFs are exposed to those organisms. The viability of the cells is usually used to determine the toxicity of the MOFs in the various cell lines. Investigations on the toxicity of MOFs and the ways in which they interact with the cells have shown that they can lead to cellular dysfunction and protection.^[230]^ The most common method of cellular internalization of MOFs occurs through endocytic processes, including processes like caveolae or clathrin‐mediated micropinocytosis, endocytosis, and phagocytosis in macrophages.^[231]^ Toxic metals interact with the cells in various ways, leading to various disease conditions. One of the main ways that MOFs interact negatively with the cells is through the induction of mitochondrial dysfunction. This occurs due to the ability of the toxic metals to interact with the electron transport chain within the mitochondria, leading to decreased synthesis of ATP by the cells, loss of membrane potential across those mitochondria, and increased production of ROS within the cells. The specific toxicity of certain MOFs for certain organs is likely related to the way in which those MOFs interact with the specific dysfunction of those organs, which is the reason for the nephrotoxicity of MOFs on the proximal tubule cells of the kidneys, for instance. Stress‐responsive mechanisms are also activated by this type of disruption, including the process of mitophagy. Mitophagy is a form of autophagy that removes impaired mitochondria from the cell. However, severe stress can lead to the impairment of autophagy, leading to the accumulation of damaged organelles and cellular debris.[^132^, ^232^]

### 6.3 Regulatory concerns

MOFs applied in biomedicine are regulated by toxicity (metal ions, organic linkers), body stability (degradation, premature release), reproducibility/manufacturability (scalability), and biodistribution/excretion (long‐term fate), as issues with any of these complicate the journey from bench to bedside.^[233]^ The existing regulations (REACH, EU; TSCA, USA) must be adapted to the specific properties of MOFs, such as fragmentation and environmental evolution. Framework size, porosity, dissolution behavior, and ligand stability are examples of the parameters that should be included in the environmental risk assessment.[^234^, ^235^]

The actual MOF system of research and industrialization displays a trade‐off between sustainability and innovation. Despite the many benefits that MOFs have for their physical properties, it is essential to review the environmental merits of these materials throughout their entire life cycle. The safety and circularity of these materials cannot be replaced by their technological capabilities. It is essential to establish these precautions before MOFs become a system entry of pilot systems into various environmental and industrial environments. By utilizing the information gathered from life‐cycle assessments and degradation studies, MOFs will have a chance to become more than just green materials but truly sustainable materials.^[236]^ The science fraternity, the industrial sector, and regulatory bodies must all work together to ensure that the incorporation of MOFs into various industrial processes is done ethically, avoiding the traps that were created by the rapid incorporation of previous technologies without considering their effects on the environment and the ecosystem as a whole. Furthermore, it is also desirable to incorporate sustainability into the MOFs themselves, not only in the applications of MOFs to various fields but also in the life cycle of MOFs as a whole. MOFs must be protected against becoming a future waste product, and such protections must be instituted in the present day before MOFs can begin to be utilized in widespread ways beyond pilot systems.^[237]^

### 6.4 Challenges in the clinical translation of smart porous MOFs

Despite having several obstacles to their development and use, the unique features of MOFs have made them the more popular choice in relation to the current trend and research in the field. MOFs can perform a variety of key characteristics within biological systems, such as biodegradability, circulation, stability, and selectivity. The potential function of these MOFs is determined by a variety of different factors.[^217^, ^238^, ^239^] However, due to the limited number of studies comparing MOFs to other types of drugs or treatment methods, the clinical application of MOFs is not yet entirely understood.^[240]^ The formulation of MOFs proves to be significantly challenging because of their tendency to degrade and unequal distribution. Even though there are stability challenges in modifying the MOFs' surfaces, the initial results are promising. However, it is crucial to analyze the synthesis of these MOFs to determine how the surface was modified and the resulting properties of the MOFs.^[50]^ It is essential to overcome these challenges to improve the effectiveness of MOFs in biological applications. Another essential aspect to consider with MOFs is their biocompatibility and toxicity. There is a lack of findings concerning the in vivo toxicity and misdistribution of several newly synthesized MOFs. These parameters are essential for performing biocompatibility tests on these novel MOFs. Several in vitro studies on the toxicity of MOFs have used various cell lines. However, these results are challenging to obtain owing to the various in vitro conditions used in these studies.^[241]^ Furthermore, it is also essential to study the interaction of these MOFs with biological systems and their metabolic processes before performing any in vivo or preclinical tests on these novel materials.^[240]^ In order to advance the research on MOFs, a uniform synthesis methodology and attributes for MOFs from several research organizations around the world are required, as well as a standardization of the protocols for MOFs synthesis, characterization, data collection, and screening processes.^[128]^ Such a standardization of synthesis procedures will allow researchers to continue to perform research on these MOFs to ensure their safety and efficacy for their biological applications. Beyond the synthesis of these MOFs, it is also critical to understand the mechanism of action of these nanostructured materials within biological systems. It is also critical to analyze the techniques that will ensure the sustainable and safe use of these MOFs within biological systems. Additionally, it is important to perform research on the environmental impact of MOFs.^[242]^

### 6.5 Future perspectives: Engineering roadmap toward clinically translatable smart porous nanoplatforms

Going forward, the focus will likely shift toward engineering advances in MOFs as a means of improving their biological translatability, rather than into expanding the range of biological applications that can be developed based upon these nanomaterials. The next generation of MOFs will likely evolve from multifunctional MOFs towards MOFs that can be utilized as targeted nanomedicine for specific biological applications.^[243]^ One of the main areas of interest is in the creation of MOFs that are responsive to multiple stimuli and pathological parameters within the body, such as pH, glutathione levels, hypoxia, enzymes, inflammatory mediators, and ROS. Unlike the current MOFs that are only responsive to a single stimulus, these types of “intelligent” nanoplatforms would allow for the targeted and specific delivery of therapeutic agents to targeted cells or locations within the body.^[128]^

Another interesting trend for the future of MOFs is the incorporation of biologically inspired engineering strategies. For instance, engineering MOFs that incorporate cell membranes, extracellular vesicles, or even that incorporate biological components into their structure will allow nanomedicine to avoid immune system detection to specifically target certain cells or tissues, and to enhance the specificity of biologically active MOF‐based therapies.^[244]^ Finally, the integration of AI, machine learning, and computational sciences into the field of MOF engineering is another interesting trend for the future of these materials. For instance, utilizing AI and machine learning models to discover new MOFs with desired properties would allow for the rapid creation of new types of MOF‐based nanotherapeutics.^[245]^ Data‐driven approaches to predict the stability of MOFs, their pore architectures, biodegradability, drug‐loading efficiencies, and biological performance will accelerate the development of these MOFs and allow for their rational design.^[246]^

Future smart MOFs are also likely to incorporate increasing levels of sophistication in their theranostic capabilities. Such MOFs may enable personalized medicine strategies for treating individuals based on their characteristics. Furthermore, efforts must also be made to produce these MOFs in a manner that is manufacturable, reproducible, safe, and cost‐effective. Each of these factors is equally as important as their potential to provide therapeutic benefits to the patients who are to be treated with these MOFs.^[247]^ Overall, then, the future of smart MOFs is likely to include the incorporation of intelligence into the MOFs themselves, the need for personalized medicine with those MOFs, and the need to be able to manufacture those MOFs in a cost‐effective manner. These advances will transform MOFs from nanocarriers with high efficiency to clinically applicable MOFs with multiple functions for diagnostics, therapeutics, regenerative medicine, and nanomedicine.^[248]^

## 7 CONCLUSION

MOFs have the potential to transform several different areas of medical technology. For instance, MOFs can be used in the delivery of drugs, imaging technology in medicine, tissue engineering, and even antimicrobial medicines. The properties of MOFs make them ideal candidates for precision medicine therapies for individual patients with various conditions. Additionally, their large surface area allows drugs to be delivered in a controlled manner to patients' cells. Furthermore, MOFs can be engineered to deliver drugs to specific areas of a patient's body. MOFs have also been utilized in bioimaging to enhance the contrast between those tissues and the surrounding medium for improved imaging. MOFs can also be utilized as scaffolds for tissue engineering applications, as they enable cells to grow on their surfaces. Additionally, they can contain substances that enhance the outcomes of tissue engineering treatments. Furthermore, MOFs have the ability to efficiently deliver therapeutic compounds to targeted areas of the body, suggesting their use in future antimicrobial or antiviral treatments. Finally, because MOFs are biodegradable and biocompatible with the human body, the risk of toxicity is inhibited over time. Their adaptability allows for their use in producing customized distributions of the medication with minimal detrimental impact on patients. Though many breakthroughs have been made in the field, some of the challenges of MOFs, such as potential toxicity, environmental impact, and the even distribution of the medication to patients, still exist. AI and machine learning have the potential to greatly reduce the time necessary to develop MOFs that interact with biomolecules in particular ways, allowing for the creation of MOF nanomaterials with desired features for specific applications. One of the significant potentials of MOFs in the area of personalized medicine is their ability to be tailored to the genetics of individual patients and to be administered to those patients through a specific route of delivery of a specific drug. Ongoing research regarding MOFs can help to better optimize the properties of these MOFs to ensure their sustainability and safety, and sustainability. Additionally, research beyond the medical field in which MOFs can be applied also has the potential to lead to breakthroughs in the applications of MOFs to medicine and other fields. Overall, then, MOFs and MOF‐based nanomaterials have the potential to make even more significant contributions to the fields in which they are applied in the future. From the passive carriers to the smart nanoplatforms, there is a paradigm shift taking place in the world of nanomedicine. The future of MOFs in nanomedicine will not rely on the discovery of new MOF structures but engineering them to meet the requirements of biological responsiveness, immune compatibility, pharmacokinetics, and regulatory requirements.

## CONFLICT OF INTEREST STATEMENT

The authors declare no conflicts of interest.

## Data Availability

Data sharing not applicable to this article as no datasets were generated or analysed during the current study.
